# Prevalence of hypervirulent and carbapenem-resistant *Klebsiella pneumoniae* under divergent evolutionary patterns

**DOI:** 10.1080/22221751.2022.2103454

**Published:** 2022-08-05

**Authors:** Dongxing Tian, Xiao Liu, Wenjie Chen, Ying Zhou, Dakang Hu, Weiwen Wang, Jinzuan Wu, Qing Mu, Xiaofei Jiang

**Affiliations:** a Department of Clinical Laboratory, Huashan Hospital, Fudan University, Shanghai, People’s Republic of China; b School of Pharmacy, Fudan University, Shanghai, People’s Republic of China; c Department of Infectious Disease, Huashan Hospital, Fudan University, Shanghai, People’s Republic of China; d Department of Clinical Laboratory Medicine, Shanghai Pulmonary Hospital, Tongji University School of Medicine, Shanghai, People’s Republic of China; e Department of Clinical Laboratory, Pingyang Hospital of Wenzhou Medical University, Wenzhou, People’s Republic of China

**Keywords:** Hypervirulent, conjugation, *oriT*, genome evolution, carbapenem resistance

## Abstract

K1/K2 hvKP strains acquire carbapenem-resistance plasmids, known as CR-hvKp, and carbapenem-resistant *Klebsiella pneumoniae* (CRKP) strains obtain virulence plasmids, recognized as hv-CRKP. The two different evolution patterns of hypervirulent combined carbapenem-resistant *Klebsiella pneumoniae* may lead to their different prevalence in hospitals. Our study aimed to investigate the prevalence of hv-CRKP and CR-hvKp strains and to analyze factors influencing their evolution and prevalence. We collected 890 *K. pneumoniae* genomes from GenBank and 530 clinical *K. pneumoniae* isolates from nine hospitals. Our study found that hv-CRKP strains were more prevalent than CR-hvKp strains and both were dominated by *bla*_KPC-2_ gene. The *bla*_KPC-2_-carrying plasmids could mobilize non-conjugative virulence plasmids from hvKp strains to CRKP strains. The conserved *oriT* of virulence plasmids and the widespread of conjugative helper plasmids were potential factors for the mobilization of non-conjugative virulence plasmids. HvKp strains with KPC plasmid could hardly simultaneously exhibit hypervirulence and carbapenem resistance as CRKP strains with virulence plasmid, and we found that *rfaH* mutation reduced capsular synthesis and increased carbapenem resistance of the CR-hvKp strain. In summary, this study revealed that hv-CRKP strains were more suitable for survival in hospital settings than CR-hvKp strains and the widespread conjugative KPC-producing plasmids contributed to the emergence and prevalence of hv-CRKP strains.

## Introduction

*Klebsiella pneumoniae,* an opportunistic pathogen, exhibits significant adaptations across diverse habitats. Its successful persistence in clinical settings may be attributed to antimicrobial resistance and virulence. The hypervirulent *Klebsiella pneumoniae* (hvKp), which causes invasive infections in the communities, exhibits high pathogenicity, but limited resistance to antibiotics [1]. In contrast, the nosocomial-acquired classic *K. pneumoniae* has limited virulence potential but tends to acquire resistance to multiple antibiotics [2]. Such diverse genetics and phenotypes have been present for a long time [3].

Recently, the continuous evolution of plasmids of hypervirulence or carbapenem resistance has led to the emergence of hypervirulent and carbapenem-resistant *K. pneumoniae* [4,5]. The process may follow three evolutionary paths: K1/K2 hvKP strains acquired carbapenem-resistance plasmids, known as CR-hvKp [6]; acquisition of pK2044-like virulence plasmids by carbapenem-resistant *Klebsiella pneumoniae* (CRKP) strains, recognized as hv-CRKP [4]; the convergence of virulence and carbapenem resistance in a single plasmid, carried by hvKp or CRKP strains [7,8]. ST23-CR-hvKp and ST65-CR-hvKp are the two most reported CR-hvKp strains. In China and Singapore, most CR-hvKp strains are found to produce KPC carbapenemase, while in America, UK and Japan, other carbapenemases, such as NDM, IMP, OXA-48 et al., are dominant in CR-hvKp strains [9]. Besides, CR-hvKp strains producing two types of carbapenemases are also found [9]. In contrast, hv-CRKP strains are mainly reported in China with some indications of an epidemic. ST11-hv-CRKP producing KPC carbapenemase has an overwhelming dominance of CR-hvKp strains [10]. The *bla*_KPC-_positive plasmids are commonly IncFII conjugative plasmids with the ability to transfer between different strains and even between different species [11,12]. It seems easier for hvKp strains to obtain KPC plasmids than CRKP strains to obtain non-conjugative virulence plasmids. Thus, CR-hvKp is supposed to be more widely spread than hv-CRKP but this was not the case. Several outbreaks of infection caused by hv-CRKP strains, particularly ST11-hv-CRKP, have been reported in Chinese hospitals [4,13]. By comparison, CR-hvKp infections are less frequently reported and appear to cause fewer hospital outbreaks, especially in China. However, the factors affecting the emergence and prevalence of hv-CRKP and CR-hvKp strains in hospitals remain unclear.

The hv-CRKP strain harbours a pK2044-like virulence plasmid carrying *iutA-iucABCD* and *rmpA* or *rmpA2* [14]. Such virulence plasmids are unable to self-transfer due to the absence of transfer modules. This raises the question of how the virulence plasmid is transferred from hvKp strains to CRKP strains. The hybrid or fusion plasmids with both a conjugative system and virulence clusters may have the ability to transfer to other strains [15]. Non-conjugative virulence plasmids of *K. pneumoniae* can also be mobilized by other self-transferable plasmids, as previously described [16]*,* but require more supporting evidence.

Capsule Polysaccharide (CPS) production is one of the most important virulence factors of bacteria, which is involved in resistance to complement-mediated killing and neutrophil-mediated phagocytosis [17]. Several studies indicated that change in CPS production also affects the susceptibilities of some antibiotics, such as polymyxin, carbapenem, and tigecycline [18–20]. RfaH acts as a transcriptional antiterminator and is required for capsule production and resistance to complement-mediated serum killing [21]. whether *rfaH* mutation plays a role in the carbapenem resistance of hypervirulent and carbapenem-resistant *K. pneumoniae* needs further investigation.

Our study investigated the prevalence of CR-hvKp and hv-CRKP strains based on GenBank genomes and multicenter clinical *K. pneumoniae* isolates, confirmed the important role of conjugative KPC-producing plasmids in the emergence and prevalence of hv-CRKP strains in hospitals, the role of *rfaH* in the maintenance of carbapenem resistance and hypervirulence, and analyzed factors affecting the prevalence of CR-hvKp and hv-CRKP strains in hospital settings from the evolution patterns, carbapenem resistance, and hypervirulence.

## Methods

### Strains and definitions

As of 31 May 2021, 890 sequenced *K. pneumoniae* genomes, together with 3265 plasmids, were available in GenBank, and the characteristics of these genomes are listed in Table S2. The plasmids containing the *rmpA* (or *rmpA2*) and the *iucABCD-iutA* were defined as putative virulence plasmids because the presence of these genes was sufficient for the host strain to exhibit a degree of hypervirulence, as previously reported [22,23]. Besides, 530 *K. pneumoniae* clinical isolates were previously collected from nine hospitals in seven provinces from January 2017 to February 2018, and the strain details were provided in Supplementary Methods.

The strains harbouring the putative virulence plasmids were defined as hvKp (Table S3); the strains harbouring carbapenem resistance genes, such as *bla*_KPC-2_, *bla*_NDM-1_, and *bla*_OXA-48_, were defined as CRKP (Table S4); the strains evolved from CRKP acquiring putative virulence plasmids were defined as hv-CRKP; the strains evolved from hvKp acquiring carbapenem-resistant plasmids were defined as CR-hvKp (Table S5). The CRKP and hvKp were classified according to the study by Lam et al. [24]. They analyzed 9705 *Klebsiella* genomes and 174 unique convergence events to determine the order of acquisition which was trivially assigned as virulence then resistance, or resistance then virulence, respectively. Those strains that were not assigned to either group were defined as “unassigned” clones. The sequence type and K_locus of the strains, antimicrobial resistance genes and virulence genes were determined using *Kleborate* (http://github.com/katholt/Kleborate) [24].

### Antimicrobial susceptibility test

Microdilution method was performed to determine the minimum inhibitory concentration (MIC) of the following antimicrobial susceptibilities: amikacin, ampicillin/sulbactam, piperacillin/tazobactam, cefazolin, cefuroxime, ceftriaxone, cefepime, cefoperazone/sulbactam, chloramphenicol, gentamicin, levofloxacin, trimethoprim/sulphamethoxazole, meropenem, tigecycline, and nitrofurantoin. The results were interpreted according to the Clinical and Laboratory Standards Institute [25].

### Plasmid conjugation experiment

CRKP strain HS11286, JS187, and hvKp strain NTUH-K2044 used in this study were completely sequenced before (GCA_000240185.2; GCA_002848565.1; GCA_000009885.1) [14,26]. For the first-round plasmid conjugation experiment, carbapenem-resistant HS11286 was used as a donor and tellurite-resistant NTUH-K2044 was a recipient. 200 μL of donor cells and 800 μL of recipient cells in the logarithmic phase were mixed and inoculated on the LB agar without antibiotics overnight at 37°C. The transconjugants were selected with the meropenem (1μg/mL) and potassium tellurite (3μg/mL), and were determined by PCR using *bla*_KPC-2_ as a marker gene. For the second-round conjugation experiment, the transconjugant NTUH-K2044-pKPHS2 obtained above was used as a donor, and kanamycin-resistant *K. pneumoniae* JS187 and sodium azide-resistant *E. coli* J53 were used as recipients. The transconjugants were screened with LB agar plates containing potassium tellurite (3μg/mL) and kanamycin (150 μg/mL) if JS187 was used as a recipient, and if J53 was used as a recipient, the LB agar plates containing potassium tellurite (3μg/mL) and sodium azide (150μg/mL) were used. All plasmid conjugation experiments were repeated three times. The transconjugants were further determined by PCR using *iuc*A. PFGE, S1-PFGE, and plasmid sequencing were performed as described previously to confirm the acquisition of the plasmids by the recipient strain [27,28].

### Plasmid stability experiments

Plasmid stability experiments were used to evaluate the stability of KPC plasmids and virulence plasmids in constructed hv-CRKP and CR-hvKp strains. A single colony was inoculated into 3 mL of fresh LB broth containing corresponding antibiotics. Overnight cultures were diluted at 1:1000 in fresh LB broth without antibiotics and then subcultured every 12 h. At each passage, more than 100 colonies (N) were selected to inoculate in LB agar containing meropenem or potassium tellurite and counted the strains with plasmid-loss (n) until the 25th passage. The experiment was independently repeated three times. The ratio of plasmid-loss strains was calculated: F = n/N. Colony morphologies were also recorded. Mucoid colonies were counted as M, and non-mucoid colonies were as NM, as presented in Figure S1.

### CPS production quantification

The uronic acid content was quantified to evaluate CPS production. 500 μL of overnight bacterial culture was mixed with 100 μL 1% Zwittergent 3–14 (dissolved in 100 mM citric acid buffer) and incubated at 50°C for 20 min. After centrifugation at 13,000 g for 5 min, took 300 μL of supernatant to a new EP tube, added 1.2 mL of absolute ethanol to precipitate (final concentration 80%), and placed on ice for 20 min. After another centrifugation at 13,000 g for 5 min, the precipitation was the capsular polysaccharide. All subsequent steps were followed as previously described [29].

### Quantitative siderophores production assay

The relative quantitative siderophore production was assayed via Chrome Azurol S (CAS) Assay as described in our previous study [30]. Siderophore units (Su) were used to quantify the siderophore production [31]. Each assay was performed in duplicate and repeated three times independently.

### Mice and larvae in vivo infection models

*G. mellonella* larvae used in this study weighed about 200∼400 mg and were purchased from the Tianjin Huiyude Biotech Company. The infection experiments were performed as described in our previous study [30], except that the bacterial inoculum employed was adjusted to 1×10^5^ CFU. The experiments were repeated three times.

Six-week-old male BALB/c mice were purchased from the Shanghai Medical Laboratory Animal Centre (Shanghai, China). The infection experiments were performed as described in our previous study [30], except that the bacterial inoculum employed was adjusted to 5×10^6^ CFU. The experiments were repeated twice. Survival curves were generated by Prism 8. Animal ethics approval was obtained from the Animal Ethics Committee of Huashan Hospital of Fudan University.

### Construction of plasmids and gene knockout strains

Plasmid pACYC-Hyg, pACYC-Hyg-oirT_pK2044_, and pACYC-Hyg-rfaH were constructed NEBuilder HiFi DNA Assembly Cloning Kit (NEB), following the manufacturer’s protocol. The linearized vector and inserted PCR products were amplified using Q5 High-Fidelity 2X PCR Master Mix (NEB) and further purified. Plasmid pACYC-Hyg and pACYC-Hyg-oirT_pK2044_ were then transformed into HS11286 electrocompetent cells to obtain HS11286-pACYC-Hyg and HS11286-pACYC-Hyg-oriT_pK2044_. Plasmid pACYC-Hyg-rfaH was used to complement the *rfaH* mutated or knockout strains.

The gene knockout strains NTUH-K2044-ΔoriT-pKPHS2, NTUH-K2044ΔmagAΔrfaH-pKPHS2, and RJF293-ΔrfaH-pKPHS2 were constructed using the λ-*Red* homologous recombination system as previously reported [32]. The primer sequences are presented in Table S6. The derivatives of strains and plasmids were summarized in Table S7.

### Analysis of *oriT* and TraM of sequenced plasmids

The *oriT* sequences of pK2044 and pKPHS2 were predicted in oriTfinder with the default parameter settings. The oriT_pK2044_ was used as a query against sequenced virulence plasmids by BLASTN to identify those plasmids carrying specific *oriT* sequences (identities≥95%, coverages ≥95%). The *oriT* primer was used for PCR amplification to screen *oriT*-carrying clinical hvKp strains.

The TraM proteins and TraM-carrying *Enterobacteriaceae* plasmids were identified by BLASTP and TBLASTN searches (identities ≥ 30%, coverages ≥ 90%, limited to *Enterobacteriaceae* organisms) using the TraM_pKPHS2_ protein (accession: AEW92155.1). The TraM proteins were then performed a cluster analysis by mmseq2 (cov-mode 1-c 0.9–min-seq-id 0.9), and the representative sequences were extracted to perform multi-sequence alignments by Muscle. The unrooted phylogenetic tree was generated by MEGAX using neighbour-joining method [33]. The output Newick file was visualized in iTOL (https://itol.embl.de/).

### Statistical analysis

Prism 8 (GraphPad Software, San Diego, CA) was used to calculate the significance with Student’s *t*-tests and log-rank test.

## Results

### Prevalence of hv-CRKP and CR-hvKp strains

**Figure 1.**
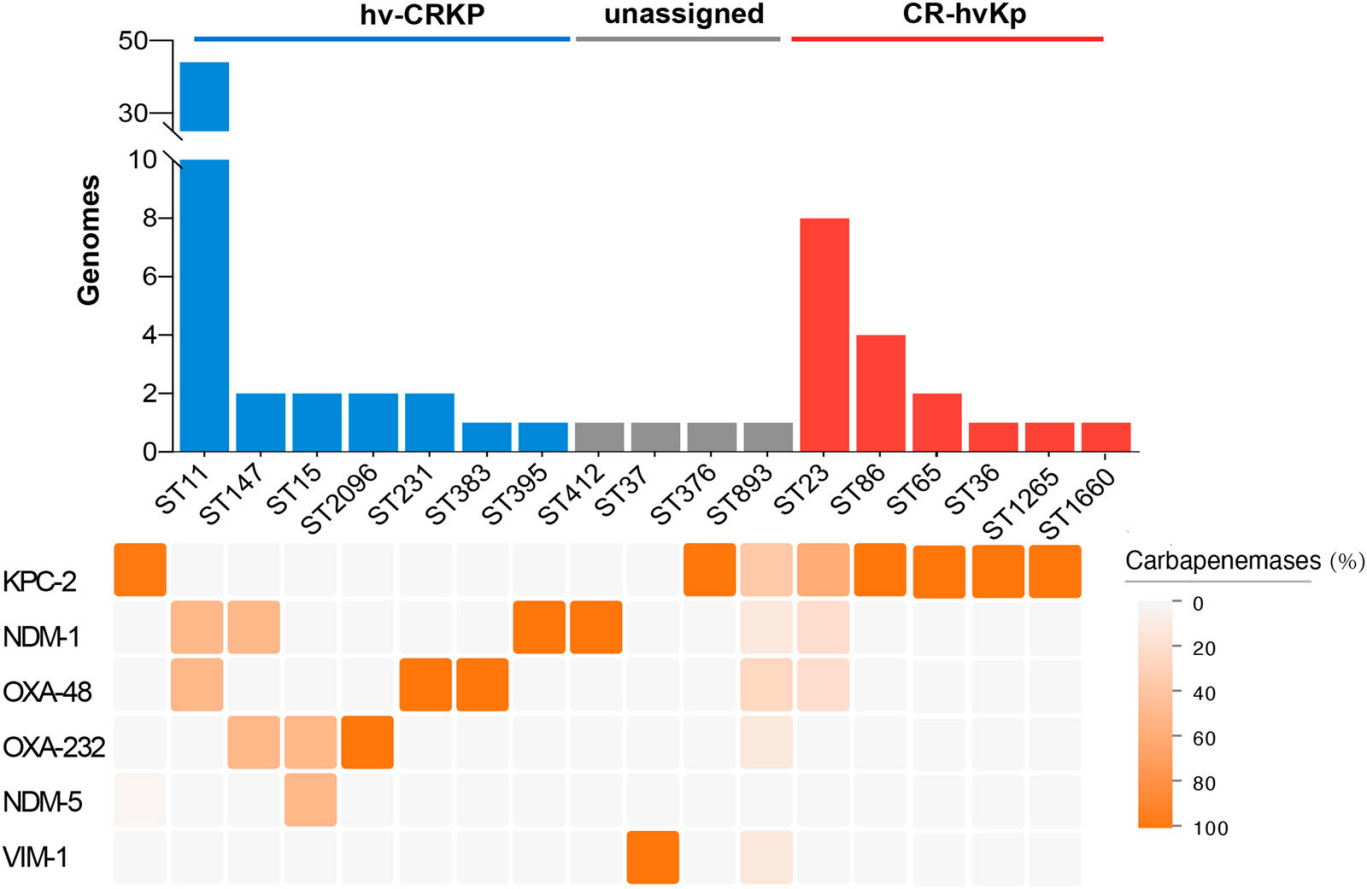
The prevalence of hv-CRKP and CR-hvKp strains. Distribution of carbapenemases is depicted as below. Intensity of box shading indicates the proportion of genomes harbouring different carbapenemases (orange), as per inset legend.

478 CRKP strains and 168 hvKp strains were identified among 890 *K. pneumoniae* genomes. Among 478 CRKP strains, KPC carbapenemase was the most detected (54.2%). The strains which belonged to both CRKP and hvKp accounted for 8.3% (Table S5). Furthermore, 71.6% of them were recognized as hv-CRKP, mainly composed of ST11-hv-CRKP harbouring *bla*_KPC-2_, while 23.0% of them were considered as CR-hvKp strains, which were most common to ST23-CR-hvKp (Figure 1). KPC was also the most detected in CR-hvKp strains (Figure 1).

To be more representative and convincing, we collected 530 multicenter clinical *K. pneumoniae* isolates. A total of 227 CRKP strains and 171 hvKp strains were identified, of which 34 strains belonged to both CRKP and hvKp (Figure S2). Among them, 28 isolates were hv-CRKP and only 6 isolates were CR-hvKp. Similarly, all hv-CRKP and CR-hvKp strains harboured *bla*_KPC-2_.

Overall, the hv-CRKP strains were more prevalent than CR-hvKp strains in hospitals, and both were dominated by *bla*_KPC-2_, indicating that plasmids carrying *bla*_KPC-2_ may play an important role in the evolution of hv-CRKP and CR-hvKp strains.

### KPC-producing plasmids mobilize non-conjugative virulence plasmid

The genetic characteristics of strains used in this study were summarized in Table S1. We first conducted plasmid conjugation assays repeatedly under different donor and recipient ratios but none of the transconjugants of pK2044 virulence plasmid to HS11286, JS187, and J53 was obtained, confirming that pK2044 virulence plasmid was not self-transferable.

**Figure 2.**
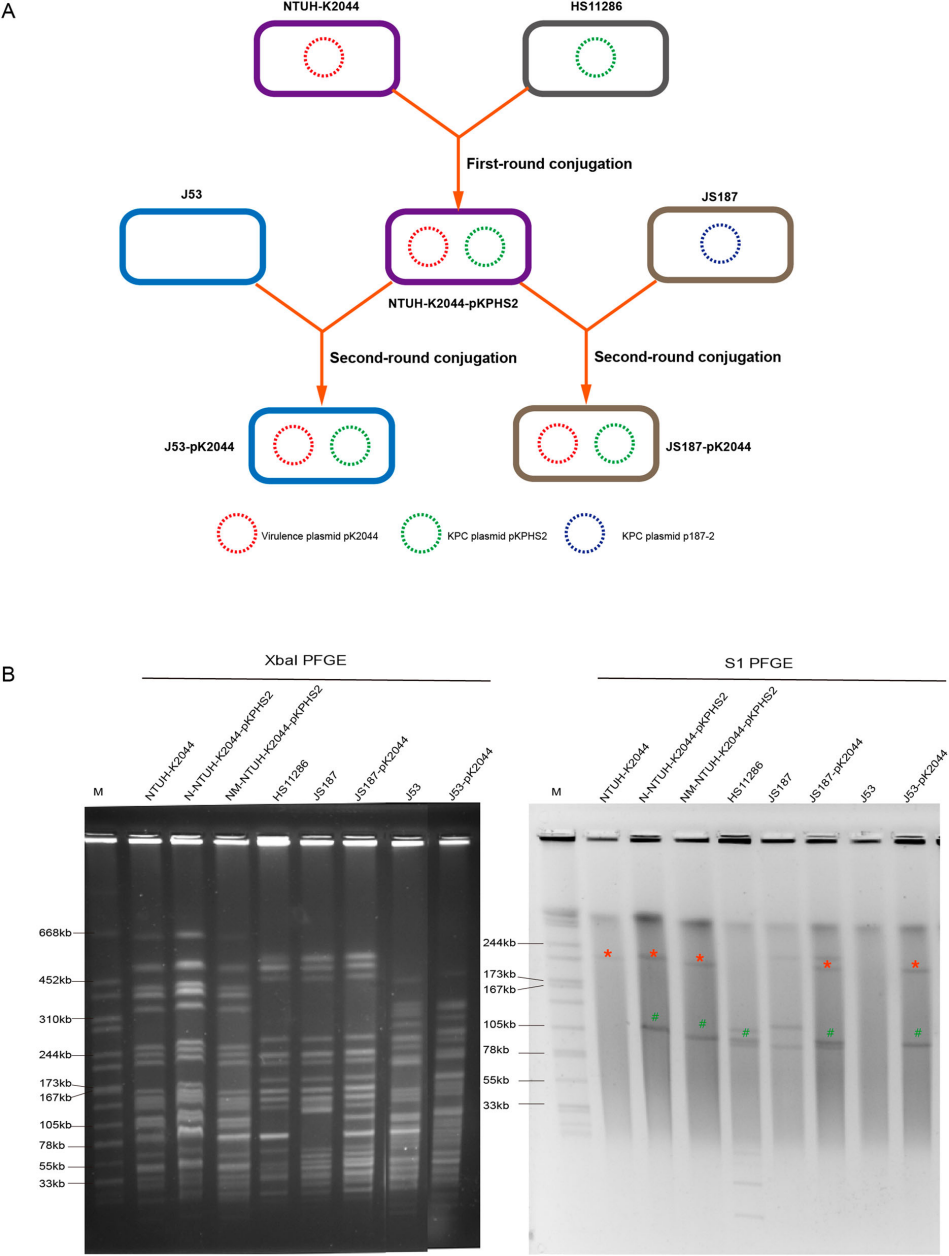
The mobilization of non-conjugative virulence plasmids by conjugative KPC plasmid. (A) Two rounds plasmid conjugation experiments simulating the transfer path of non-conjugative plasmid. (B) The confirmation of transconjugants by PFGE and S1-PFGE. *Virulence plasmid pK2044. ^#^KPC plasmid pKPHS2. The same symbol is used to represent the parental strain and its transconjugant.

Two rounds of plasmid conjugation assays were performed to simulate the transfer path of non-conjugative virulence plasmids in clinical isolates (Figure 2(A)). For the first-round conjugation experiment, the KPC plasmid pKPHS2 of HS11286 was self-transferred into hypervirulent NTUH-K2044, albeit with a low conjugation frequency (3.3×10^−7^ ± 0.8×10^−7^). PFGE, S1-PFGE, and MUMmer analysis confirmed the successful transfer of KPC plasmid pKPHS2 (Figure 2(B); Figure S3; Figure S4). We selected one of the transconjugants, NTUH-K2044-pKPHS2, for further study.

In the second-round conjugation experiment, we observed that pK2044 virulence plasmid carried by NTUH-K2044-pKPHS2 could be co-transferred with conjugative KPC plasmid to CRKP strain, and transconjugant JS187-pK2044 was obtained (conjugation frequency: 2.4×10^−8^ ± 1.9×10^−8^) (Figure 2(A)). PFGE, S1-PFGE, and MUMmer analysis confirmed the mobilization of non-conjugative virulence plasmid pK2044 by conjugative KPC plasmid pKPHS2 (Figure 2(B); Figure S3). We need to point out that three transconjugants were selected to perform PFGE and S1-PFGE experiments, and the transfer of virulence plasmid alone or plasmid infusion event was not observed (Figure S4). In addition, the native plasmid p187-2 of JS187 was lost when plasmid pKPHS2 was acquired (Figure 2B; Figure S3). p187-2 and pKPHS2 were both *bla*_KPC-2_-positive IncFII plasmids, and the plasmid incompatibility resulted in the two plasmids could not reside in the same cell. Therefore, transconjugant JS187-pK2044 had five plasmids, which were p187-1, p187-3, p187-4, pKPHS2, and pK2044.

We also observed that non-conjugative virulence plasmid pK2044 could not only be mobilized to *K. pneumoniae* by conjugative KPC plasmid pKPHS2 but also to *E. coli* J53 (conjugation frequency: 7.8×10^−8^ ± 3.5×10^−8^) (Figure 2; Figure S3; Figure S4). Altogether, this study successfully demonstrated that the non-conjugative virulence plasmid could be mobilized from hvKp strain to CRKP strain facilitated by conjugative KPC plasmid, and then developed into the hv-CRKP strain possessing a KPC plasmid and a virulence plasmid.

### Transfer potential of mobilizable virulence plasmids

**Figure 3.**
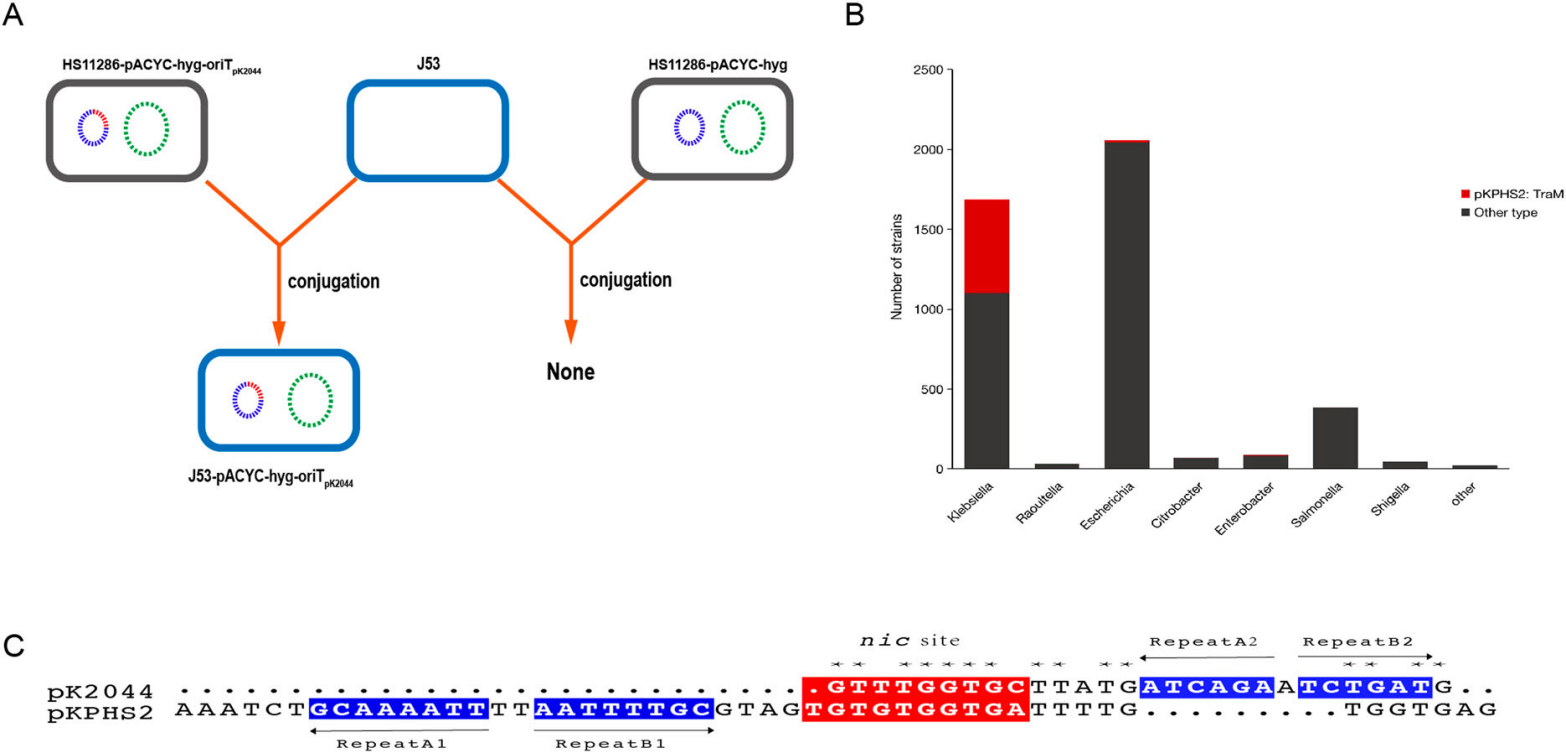
The transfer potential of mobilizable virulence plasmids. (A) The mobilization of mimic virulence plasmid by conjugative KPC plasmid pKPHS2. (B) The distribution of TraM_pKPHS2_-carrying plasmids in *Enterobacteriaceae*. (C) *nic* sites of pKPHS2 and pK2044. Alignment was generated with the MUSCLE sequence alignment tool.

We predicted the *oriT* sequences of the virulence plasmid pK2044 and conjugative plasmid pKPHS2 by oriTfinder (Figure S5A). Two predicted T4CPs were also presented in virulence pK2044 (Figure S5A and S5C). The *nic* site of pK2044 and pKPHS2 had a certain similarity, but the IR sequences are quite different (Figure 3(C)). A study on *Staphylococcus aureus* proposed that conjugative plasmid mobilization was found to be specific for plasmids carrying matching IR2 sequences [34]. To test whether the predicted *oriT* sequence was essential for the mobilization of virulence plasmid, we constructed the *oriT*-knockout strain of NTUH-K2044-ΔoriT-pKPHS2 to perform plasmid conjugation assay with the donor strain JS187 and J53, and the transconjugant was not obtained. Moreover, the mimic virulence plasmid pACYC-hyg-oriT_pK2044_ which carried the *oriT* sequences of pK2044 was successfully transferred to *E. coli* J53, while the empty vector pACYC-hyg was not able to be mobilized (Figure 3(A)). Together, these experiments indicated that the specific *oriT* sequence was essential for the mobilization of non-conjugative virulence plasmids, and carriage of this *oriT* by a normally non-mobilizable plasmid conferred the ability to be mobilized by conjugative KPC plasmid pKPHS2. Moreover, we observed that 94.2% of virulence plasmids in GenBank and 93.5% of clinical hvKp strains had the conserved oriT_pK2044_ sequence.

Relaxase-*oriT* recognition often involves “relaxosome accessory factors” (RAFs), and the characterized RAF in F and F-like plasmids of Gram-negative bacteria is TraM, whose N-terminal domain is involved in sequence-specific DNA recognition [34,35]. Our results showed that a total of 256 different TraM types were identified and predicted conjugative helper plasmids which were 100% identical to TraM_pKPHS2_ were widespread in *K. pneumoniae*, and these helper plasmids can also be detected in other *Enterobacteriaceae* bacteria (Figure 3(B) and Figure S5B). In summary, the conserved *oriT* sequence in virulence plasmids and the widespread of conjugative helper plasmids, such as KPC-producing plasmids, were potential factors for the mobilization of non-conjugative virulence plasmids and contributed to the emergence of hv-CRKP strains.

### Carbapenem resistance and virulence of CR-hvKp

The “CR-hvKp” strain NTUH-K2044-pKPHS2 obtained in the first-round plasmid conjugation experiment possessed a virulence plasmid and a KPC plasmid. However, NTUH-K2044-pKPHS2 strain did not show sufficient carbapenem resistance (meropenem MIC = 2 μg/mL), although acquired resistance to other β-lactam antibiotics (Table 1). NTUH-K2044-pKPHS2 strain was still sensitive to aminoglycoside, quinolones, chloramphenicol, and tigecycline (Table 1).

**Table 1.** Antimicrobial susceptibilities of strains and their transconjugants.

Strains	STs	Bacterial species	MIC (µg/mL)^a^															
			AMK	SAM	TZP	CZO	CXM	CRO	FEP	CSL	CHL	GEN	LVX	SXT	MEM	IPM	TGC	NIT
NTUH-K2044	ST23	*K. pneumoniae*	<8	8/4	<8/4	1	16	<0.25	<2	<4/2	32	<1	<0.5	<0.25/4.75	<0.25	<0.25	1	64
HS11286	ST11	*K. pneumoniae*	>128	>64/32	>256/4	>16	>64	>8	16	128/64	>64	>32	2	>8/152	32	64	<0.5	256
JS187	ST11	*K. pneumoniae*	>128	>64/32	>256/4	>16	>64	>8	>64	128/64	>64	>32	8	>8/152	64	64	<0.5	256
J53	-	*E. coli*	<8	4/2	<8/4	1	8	<0.25	<2	<4/2	8	<1	<0.5	<0.25/4.75	<0.25	<0.25	<0.5	<8
M-NTUH-K2044-pKPSH2^b^	ST23	*K. pneumoniae*	<8	>64/32	32/4	>16	>64	>8	<2	8/16	16	<1	<0.5	<0.25/4.75	2	2	<0.5	64
NM-NTUH-K2044-pKPSH2^c^	ST23	*K. pneumoniae*	<8	>64/32	>256/4	>16	>64	>8	<2	32/16	8	<1	<0.5	<0.25/4.75	16	32	<0.5	32
JS187-pK2044	ST11	*K. pneumoniae*	>128	>64/32	>256/4	>16	>64	>8	>64	>128/64	>64	>32	8	>8/152	64	64	<0.5	>256
J53-pK2044	-	*K. pneumoniae*	8	>64/32	32/4	>16	>16	>64	<2	16/8	8	<1	<0.5	<0.25/4.75	1	1	<0.5	<8

^a^ AMK, amikacin; SAM, ampicillin/sulbactam; TZP, piperacillin/tazobactam; CZO, cefazolin、CXM, cefuroxime; CRO, ceftriaxone; FEP, cefepime; CSL, cefoperazone/sulbactam; CHL, chloramphenicol; GEN, gentamicin; LVX, levofloxacin; SXT, trimethoprim/sulphamethoxazole; MEM, meropenem; TGC, tigecycline; NIT, nitrofurantion.

^b^ Three M transconjugants were respectively selected in 1st, 12th, and 24th passage for antimicrobial susceptibility tests. The results were combined together due to the same resistance phenotype.

^c^ Three NM transconjugants were respectively selected in 1st, 12th, and 24th passage for antimicrobial susceptibility tests. The results were combined together due to the same resistance phenotype.

KPC plasmid pKPHS2 was very stable in NTUH-K2044-pKPHS2 strains after 25 passages. But in the course of the plasmid stability experiment, we observed a very interesting phenomenon that non-mucoid colonies appeared in the third generation and almost completely became non-mucoid in the fifth generation, while wild type NTUH-K2044 remained mucoid phenotype throughout passages (Figure S6). The non-mucoid NTUH-K2044-pKPHS2 (NM-NTUH-K2044-pKPHS2) was confirmed to be not a contaminant by PFGE (Figure 2(B)). Strikingly, NM-NTUH-K2044-pKPHS2 transconjugants which were selected in the 1st, 12th, and 24th passage, showed a much higher level of resistance to carbapenems than that of M-NTUH-K2044-pKPHS2, and the MIC value of meropenem was 16 μg/ml (Table 1).

**Figure 4.**
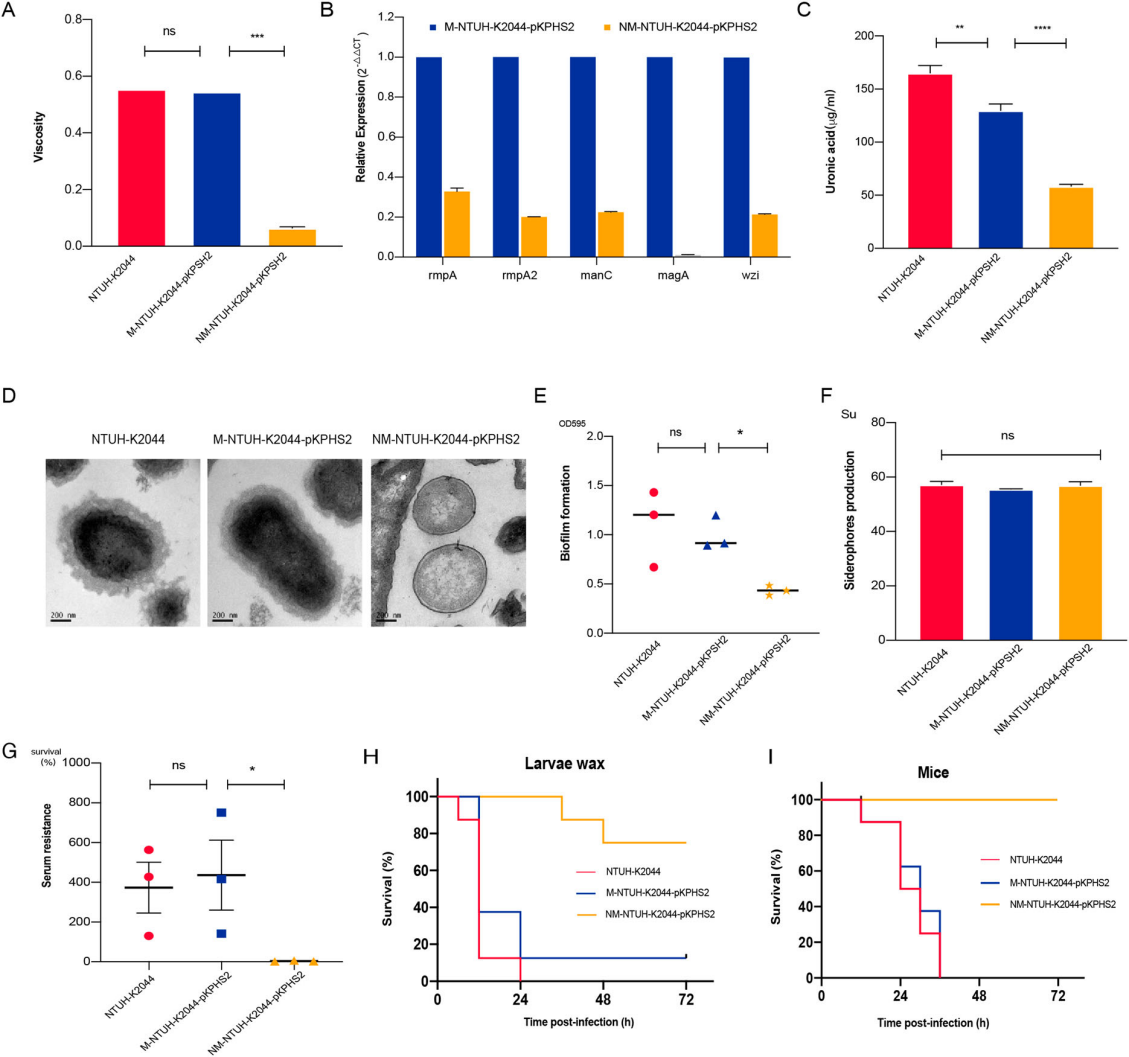
The virulence phenotypes and levels of CR-hvKp strain. (A) Mucoviscosity. (B) The relative expression of capsular synthesis genes. (C) Uronic acid. (D) Transmission electron microscopy of the capsule. (E) Biofilm formation. (F) Siderophores production. (G) Serum resistance. (H) The survival curves of infected Larvae wax. (I) The survival curves of infected mice. An unpaired two-sided Student’s t-test was performed for mucoviscosity, uronic acid, biofilm formation, siderophores production, and serum resistance. A log-rank (Mantel–Cox) test was performed for the survival curves. ***P< 0.001, **P< 0.01, *P< 0.05, ns: not significant.

Compared with M-NTUH-K2044-pKPHS2 and parental NTUH-K2044 strains, the expression of capsular biosynthesis genes (*rmpA*, *rmpA2*, *magA*, *wzi*, and *manC*), mucoviscosity, and capsular polysaccharide (CPS) production were significantly reduced in NM-NTUH-K2044-pKPHS2 (Figure 4(A–C)). Transmission electron microscopy further confirmed the capsule deficiency of NM-NTUH-K2044-pKPHS2 (Figure 4(D)). Not surprisingly NM-NTUH-K2044-pKPHS2 strain showed significantly decreased abilities of serum resistance and biofilm formation, except for the unchanged siderophore production (Figure 4(E–G)). The larvae and mice infection experiments further provided strong supporting evidence of attenuated virulence. When the larvae and mice were infected with the same bacterial inoculum, NM-NTUH-K2044-pKPHS2 exhibited significantly decreased virulence levels compared with M-NTUH-K2044-pKPHS2 and wild type NTUH-K2044 (Figure 4(H–I)).

To further verify this observation, we constructed another “CR-hvKp” strain RJF293-pKPHS2, which was formed by the conjugative transfer of KPC plasmid to K2-type hvKp strain RJF293. To our surprise, after obtaining the KPC plasmid, RJF293-pKPHS2 directly lost the hypermucoviscosity. The virulence plasmid pK2044 and KPC plasmid pKPHS2 were very stable after serial passages (Figure S6). Compared with wild type RJF293, CPS production and serum resistance were significantly reduced, and virulence levels were also decreased in the larvae and mice in vivo infection models (Figure S7). Besides, RJF293-pKPHS2 showed resistance to carbapenems (meropenem MIC = 4 μg/ml).

Taken together, our findings indicated that K1/K2-type hvKp strains obtaining the KPC plasmid were burdensome to exhibit hypervirulence and carbapenem resistance simultaneously.

### *Rfah* mutation leads to reduced capsular synthesis and increased carbapenem resistance

CPS was reported to mediate resistance to antimicrobials by limiting the interaction of the agents with membrane targets [20]. Paradoxically, as described in the previous section, NTUH-K2044-pKPHS2 increased resistance to carbapenems when CPS production was reduced. Several factors which may affect the carbapenem resistance were excluded: the expression of *bla*_KPC-2_ and the copy numbers of KPC plasmid increased slightly with no significant difference; no mutation in the *bla*_KPC-2_ gene; no mutations or deletions of porins ompK36 and ompK35; no over-production of efflux pumps (Figure S8).

**Figure 5.**
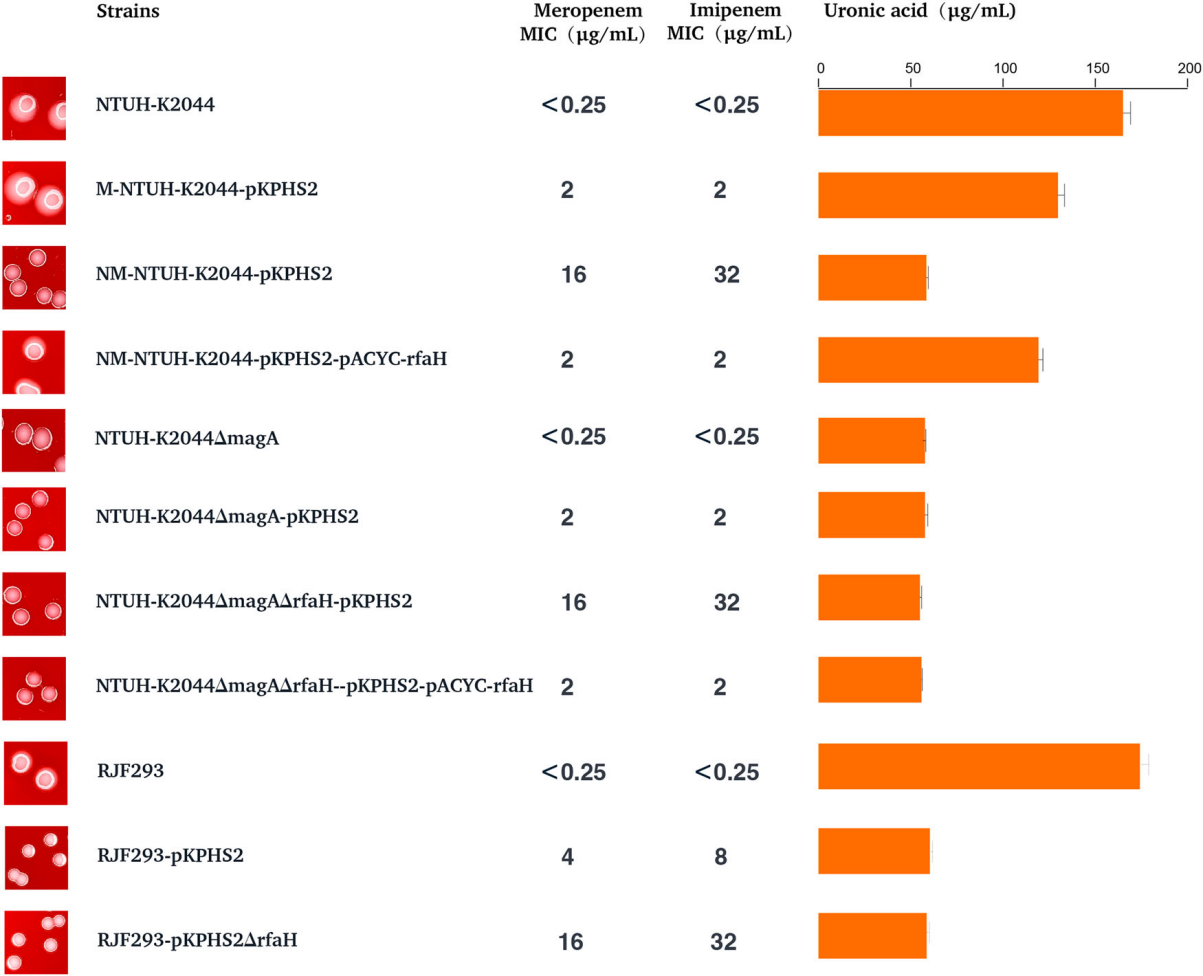
The influence of *rfaH* to carbapenem resistance and CPS production.

The single nucleotide polymorphisms (SNPs) discovery from whole-genome resequencing of NM-NTUH-K2044-pKPHS2 revealed that c.52C>T mutation *of rfaH* led to a premature stop codon (p.18Q>X). *RfaH* mutation was confirmed in another four NM-NTUH-K2044-pKPHS2 transconjugants by PCR methods and Sanger sequencing. Complementation of NM-NTUH-K2044-pKPHS2 strain with pACYC-hyg-rfaH restored CPS production and reduced carbapenem resistance simultaneously, indicating that *rfaH* mutation may involve in the CPS production and carbapenem resistance (Figure 5). In this case, it remains uncertain whether CPS reduction or *rfaH* mutation was responsible for increased carbapenem resistance. The capsule-deficient strain NTUH-K2044ΔmagA-pKPHS2 had the same MIC value of meropenem as M-NTUH-K2044-pKPHS2 (MIC = 2 μg/ml), which indicated that carbapenem resistance appeared to be independent in CPS production (Figure 5). The *rfaH* deletion strain NTUH-K2044ΔmagAΔrfaH-pKPHS2 exhibited significantly increased carbapenem resistance compared to NTUH-K2044ΔmagA-pKPHS2 (meropenem MIC increased from 2μg/ml to 16μg/ml), while *rfaH* complementation restored the MIC level of carbapenem resistance (meropenem MIC decreased from 16μg/ml to 2μg/ml), which provided strong evidence in the role of *rfaH* in affecting carbapenem resistance (Figure 5). Besides, the same trend was also observed in the *rfaH* deletion and further *rfaH* complementation of RJF293-pKPHS2 (Figure 5). These findings revealed that *rfaH* mutation would not only reduce CPS production but also increase carbapenem resistance.

It should be pointed out that *wcaJ* mutation was responsible for the reduced CPS production of RJF293-pKPHS2 according to the whole-genome resequencing analysis. Together these results indicated that such “CR-hvKp” strains would make some adjustments when obtaining the KPC plasmid, usually reducing capsular synthesis, such as *rfaH* mutation in NTUH-K2044-pKPHS2 and *wcaJ* mutation in RJF293-pKPHS2, thus contributing to an attenuation of virulence. Therefore, these “CR-hvKp” strains were not actually both hypervirulent and carbapenem-resistant.

### Carbapenem resistance and virulence of hv-CRKP

**Figure 6.**
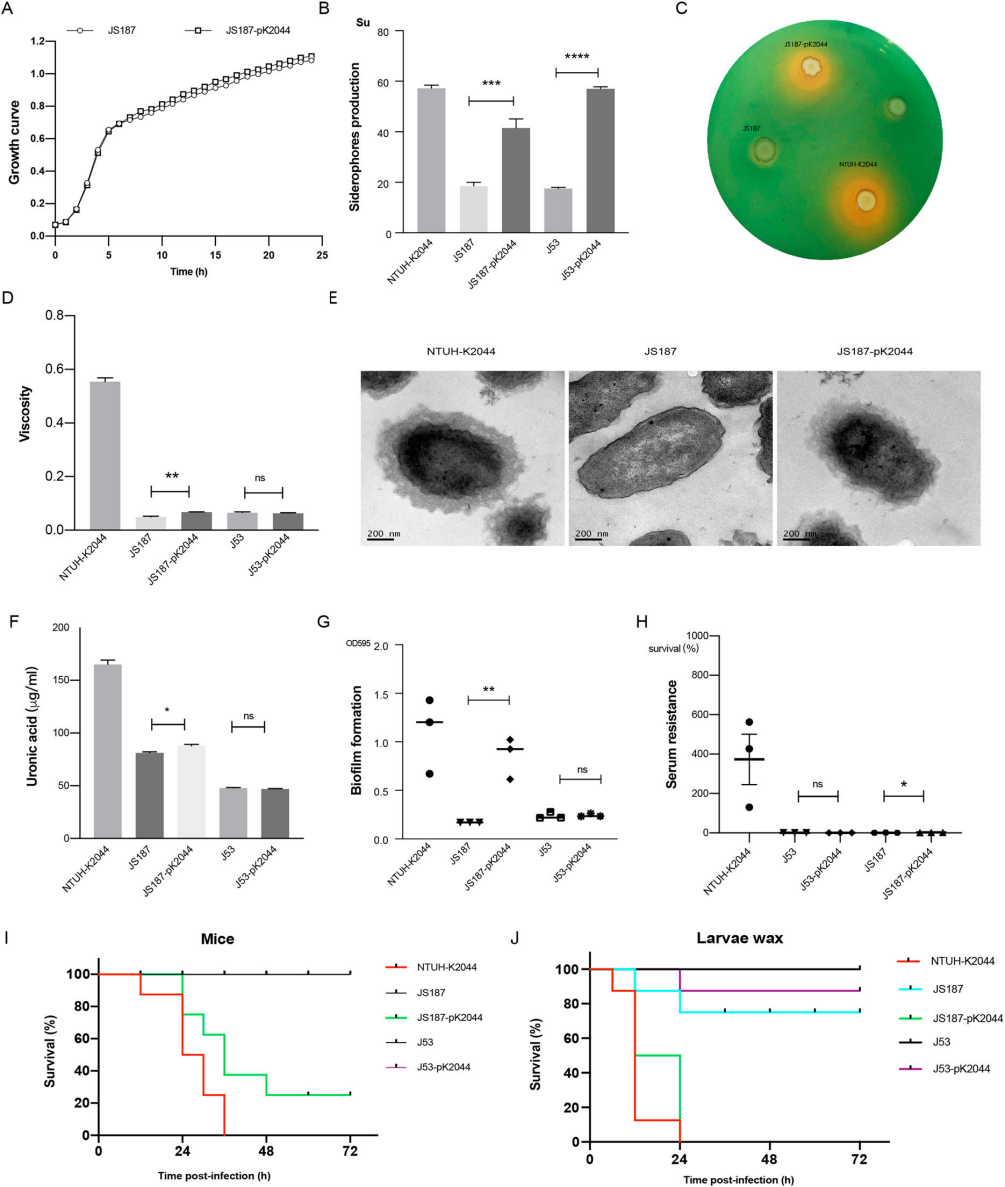
The virulence phenotypes and levels of hv-CRKP. (A) Growth curve. (B) Siderophores production quantification. (C) Siderophores production determined by CAS agar plate. (D) Mucoviscosity. (E) Transmission electron microscopy of the capsule. (F) Uronic acid. (G) Biofilm formation. (H) Serum resistance. (I) The survival curves of infected mice. (J) The survival curves of infected larvae wax. An unpaired two-sided Student’s t-test was performed for mucoviscosity, uronic acid, biofilm formation, siderophores production, and serum resistance. A log-rank (Mantel–Cox) test was performed for the survival curves. ****P < 0.0001, ***P< 0.001, **P< 0.01, *P< 0.05, ns: not significant.

The hv-CRKP strain JS187-pK2044 transconjugants showed increased mucoviscosity, siderophores production, serum resistance, and CPS production compared with parental JS187 (Figure 6). Although the virulence level of JS187-pK2044 was lower than that of hypervirulent NTUH-K2044, the much higher virulence levels of JS187-pK2044 were observed in both the larvae wax and mice infection model than that of JS187 (Figure 6). This demonstrated that the acquisition of pK2044 virulence plasmid conferred the virulence of parental JS187, albeit with the absolute level of virulence depending on the strain background. The acquisition of pK2044 did not affect the growth rate compared with parental JS187 (Figure 6). The pK2044 virulence plasmid was very stable in JS187 after 25 generations and significant colony morphology change of JS187-pK2044 was not observed during passages compared with JS187. Importantly, JS187-pK2044 transconjugants (one in 1st and two in 24th passage) still possessed a high resistance level to meropenem (MIC = 64 μg/mL) and other antibiotics (Table 1). Therefore, these results showed that hv-CRKP strains had the potential for a dual phenotype of carbapenem resistance and hypervirulence without significant burdens.

Beyond this, we observed a significant increase in siderophore production but no significant differences in mucoviscosity, biofilm formation, serum resistance, and virulence levels between *E. coli* J53 and transconjugant J53-pK2044 (Figure 6). The resistance to β-lactams was significantly increased after acquiring the pKPHS2 (Table 1).

## Discussion

Hv-CRKP and CR-hvKp are two well-characterized groups based on the distinct evolutionary pathways. In this study, we observed that hv-CRKP strains were more prevalent than CR-hvKp strains in hospitals. Lam et al. observed the opposite results that the acquisition of AMR in strains already carrying *iuc* was more identified than the acquisition of AMR by known hypervirulent clones [24]. Those strains involved in local outbreaks or large-scale spread were excluded in their study and 174 unique convergence events were finally included, which could explain the different observations. Both horizontal transfer and clonal dissemination are responsible for the widespread of CRKP strains, and the clonal dissemination is difficult to control, especially in hospitals. Therefore, our study largely indicated a true distribution of hv-CRKP and CR-hvKp strains, while the study by Lam et al. provided a comprehensive typing profile of *K. pneumoniae* with AMR and hypervirulence. In addition, our study focuses on the *K. pneumoniae* with hypervirulence and carbapenem resistance, while the study by Lam et al. analyzed other AMR genes and virulence genes. Our results also showed that KPC carbapenemase was predominated in both hv-CRKP and CR-hvKp strains, highlighting the important role of KPC-producing plasmids in the evolution of these threatening bacteria.

Virulence plasmids which specifically carried in K1/K2 hvKp strains lack conjugative transfer systems and thus are considered to be non-self-transferrable. However, the acquisition of virulence plasmids by CRKP strains has been frequently reported recently [36,37]. Our previous study found that virulence plasmids may have undergone continuous evolution and generated hybrid or conjugative plasmids [5]. A previous study reported that a fusion pLVPK-like virulence plasmid was transferred to other clinical strains [15]. However, virulence plasmids carried by ST11 hv-CRKP were not such hybrid plasmids, indicating some other underlying mechanisms. In this work, we simulated the transfer path of a non-conjugative virulence plasmid from the hvKP strain to CRKP strain or *E. coli* J53.

A mobilizable element lacking a relaxase can be mobilized by another element’s conjugation system and relaxase protein *in trans*, if it has a “mimic” of the conjugative element’s *oriT* sequence [38]. We suspect that the *oriT* of pK2044-like non-conjugative virulence plasmids could recruit the relaxosome of the KPC-producing plasmids to initiate the conjugation process, and some further compelling evidence was provided in our study. Presence of a specific *oriT*_pK2044_ in the plasmid itself is essential for the mobilization of a virulence plasmid. Our study also observed that most hvKp strains possessed this conserved *oriT*_pK2044_, indicating the mobilize potential of virulence plasmids by pKPHS2-like plasmids. TraM, the RAFs in the F and F-like plasmids in Gram-negative bacteria, is responsible for the specific recognition of *oriT* [35]. Our study revealed a rich diversity of TraM proteins, and TraM_pKPHS2_-carrying plasmids were widespread in *K. pneumoniae*. *K. pneumoniae* is an important pathogen in nosocomial infections, and CRKP strains that carried KPC-producing conjugative plasmids have been widely disseminated in hospitals. In addition, TraM_pKPHS2_-carrying plasmids were identified in other *Enterobacteriaceae* bacteria, highlighting the widespread of conjugative helper plasmids which had the potential to mobilize non-conjugative virulence plasmids and further drive the emergence and epidemic of hv-CRKP strains in the hospitals. Similar results were observed in the study by Xu et al. that virulence plasmid could be mobilized alone or co-transferred with the self-transferable IncF plasmid [16]. Their speculations were confirmed in *E. coli* model bacteria, and they focused more on the integration and resolution events. However, we did not observe the formation of hybrid plasmid during the plasmid transfer process although pK2044 and p187-2 both had the 28-bp fusion site described by Xu et al. The integration and resolution events may not occur frequently. We provided further supporting evidence using *K. pneumoniae* clinical isolates and advocated that the presence of oriTpK2044 in most non-conjugative virulence plasmids, as well as the widespread of conjugative KPC plasmids in the hospitals, largely contributed to the emergence of hv-CRKP, and hvKp strains harbouring conjugative KPC plasmids could serve as the intermediate to deliver the virulence plasmid from hvKp to CRKP. To this point, Xu et al. suggest that *E. coli* strains harbouring conjugative IncF plasmids serve as the intermediate vector to deliver the virulence plasmid indirectly from hypermucoviscous hvKP into CRKP, and our study is a good complement to their study.

Acquisition of a conjugative plasmid by hvKp strains, such as KPC-producing plasmids, is the critical step to mobilization of the non-conjugative virulence plasmids as we above described. Therefore, CR-hvKp strains are likely to evolve easier than hv-CRKP strains. Conversely, the ease of evolution of CR-hvKp does not correspond with our observations that hv-CRKP strains were in the dominance of hypervirulent and carbapenem-resistant *K. pneumoniae* in hospitals. The survival and prevalence of bacteria in hospitals is likely to be a consequence of multiple factors. Here, we have shown that K1-type hvKp strain with hypermucoviscosity did not exhibit corresponding carbapenem resistance initially after obtaining the KPC plasmid, but acquired high resistance to carbapenems when the hypermucoviscosity is lost. In comparison, K2-type hvKp strain obtaining the KPC plasmid directly lost the mucoid phenotype. CPS responsible for the hypermucoviscosity is considered a critical virulence factor [23]. Our data also showed that such “CR-hvKp” strains after the loss of hypermucoviscosity attenuated their virulence in mice and larvae in vivo. This suggests that “CR-hvKp” strains are not actually hypervirulent and carbapenem-resistant.

The assembly of CPS and formation of hypermucoviscosity are extraordinarily energy-consuming processes that are hardwired to the metabolic pulse of the cell [29]. When obtaining the KPC plasmid, “CR-hvKp” strains would make fast adjustments to reduce the energetic burden of the host. *RfaH* mutation and *wcaJ* mutation were identified in this study, leading to the reduced CPS production in K1-type “CR-hvKp” and K2-type “CR-hvKp”, respectively. It is well-established that *wcaJ* and *rfaH* involve in capsule synthesis, and its mutation affects CPS production and attenuates virulence. In this work, the complementation of non-mucoid CR-hvKp strains with wild type *rfaH* restored the levels of virulence and CPS production, confirming that the *rfaH* mutation of “CR-hvKp” strains caused the loss of hypermucoviscosity. The study by Adriana showed that in four out of eight CRKP isolates, the capsule loss in the NM variant resulted in increased susceptibilities to carbapenems, and one NM variant showed a decrease in susceptibility to carbapenems [19]. The relationship between capsular production and carbapenem resistance is uncertain. However, we observed that carbapenem resistance was significantly increased after the loss of mucoid phenotype, with MIC values of meropenem increasing from 2 μg/ml to 16 μg/ml, while resistance to carbapenem decreased significantly after *rfaH* complementation. This raises the question that what is the linkage between *rfaH* gene and carbapenem resistance. Considering that *rfaH* mutation would alter CPS production, therefore, to discriminate whether CPS production was responsible for altered carbapenem resistance in the “CR-hvKp” strains here, we knocked out *rfaH* gene from CPS-deficient strain NTUH-K2044ΔmagA-pKPHS2. The results showed that the carbapenem resistance still increased significantly, albeit with the deficiency of capsule, and *rfaH* complementation restored the levels of carbapenem resistance. Considering these data altogether, we propose that carbapenem resistance of “CR-hvKp” strains is independent of the CPS production, and highlight that *rfaH* mutation affects the carbapenem resistance. This hypothesis was further demonstrated in the K2-type “CR-hvKp” strains. The *rfaH* mutation not only results in the loss of hypermucoviscosity but also attributes to increased carbapenem resistance by a yet unknown mechanism. The defects in the O-antigen caused by *rfaH* mutation might disturb the assembly of outer membrane proteins, resulting in a decrease in carbapenem permeability and susceptibility [19].

Overproduction of CPS provides a fitness advantage for invasive infections, but at a metabolic cost [29]. It is possible that in hospital settings the metabolic burdens of elevated CPS biosynthesis and hypermucoviscosity may pose a fitness disadvantage. This cost–benefit balance between CPS biosynthesis and adequate energy source resisting antibiotic stress may explain the loss of hypermucoviscosity of “CR-hvKp” strains. Previous studies indicated that the acquisition of colistin and tigecycline resistance in hvKp strains were accompanied by reduced capsular production and decreased virulence, and significant fitness costs [18,39]. It is a large burden for the bacteria to stably exhibit hypervirulence and drug resistance, which may be a potential disadvantage for their survival in hospitals. Such “hv-CRKP” strains will be rapidly be killed by other agents, such as aminoglycosides and quinolones, due to the absence of corresponding resistance genes. Importantly, these “hv-CRKP” strains are possible as reservoirs to spread non-conjugative virulence plasmid to other clinical strains, such as CRKP strains. CRKP strains were found to have a hypervirulence phenotype after acquiring pK2044-like virulence plasmids [4]. Consistently, ST11-CRKP JS187 acquired a pK2044 virulence plasmid and then possessed an enhanced virulence phenotype and a thick capsule, exhibiting carbapenem resistance and hypervirulence. It is pointed out that pK2044 virulence plasmid stably persists in both JS187 and J53, a significantly increased virulence was not observed in J53-pK2044 as that in JS187-pK2044. Different genetic backgrounds between *K. pneumoniae* and *E. coli* may explain the deficient expression of the corresponding virulence genes in *E. coli*. More importantly, hv-CRKP strain still remained resistant to many antimicrobials, which facilitated their survival in hospitals.

Altogether, the ease of evolution of “CR-hvKp” strains cannot counteract the disadvantages of their survival in hospitals. The acquisition of conjugative KPC-producing plasmids by hvKp strains enables the mobilization of non-conjugative virulence plasmids to CRKP strains and thus contributes to the emergence of CR-hvKp strains. These CR-hvKp strains not only have the potential to adapt to survive in the antibiotic stress environment, but also act as “super-spreaders” of virulence plasmids to spread to more CRKP strains. The clonal dissemination of CRKP and hv-CRKP strains further aggravate the epidemic of hv-CRKP strains in hospitals. Currently, nosocomial infections caused by CRKP remain an unresolved issue, and the self-transferability of the KPC-producing plasmids is the main culprit for the wide dissemination. As described in this study, conjugative KPC-producing plasmids also act as an important contributor to the emergence and prevalence of hypervirulent and carbapenem-resistant *K. pneumoniae* in hospitals.

## Data access

The plasmid sequences of transconjugants were submitted to GenBank (MZ475697, MZ475698, MZ475700, MZ475701, MZ475702, MZ475703, MZ4757004, MZ475709, and MZ475710). The bam files of whole-genome resequencing were deposited at the NCBI Sequence Read Archive (SRA) with the BioProject accession number PRJNA819844.

## Supplementary Material

Supplemental Material
