# Supplementary material for: Prevalence of hypervirulent and carbapenem-resistant Klebsiella pneumoniae under divergent evolutionary patterns
Source: Emerg Microbes Infect. 2022 Aug 5;11(1):1936–49. doi: 10.1080/22221751.2022.2103454 (PMC9359173; doi:10.1080/22221751.2022.2103454)
Supplement: Supplemental Material [file TEMI_A_2103454_SM8825.zip › Supplementary_Materials/supplemental materials.pdf]

Supplementary Methods

Multicenter clinical *K. pneumoniae* strains

530 clinical *K. pneumoniae* isolates included 271 isolates from Huashan Hospital of Fudan University, 60 isolates from the Sixth Hospital of Shanxi Medical University, 52 isolates from Taizhou Municipal Hospital, 43 isolates from Kunming Yan'an Hospital, 31 isolates from First Affiliated Hospital of Xiamen University, 29 isolates from Jinshan Hospital, 27 isolates from Shandong Provincial Hospital, and 17 isolates from First Affiliated Hospital of Guangxi Medical University. The sequence types and virulence genes were previously determined in other studies [1-4]. This study supplemented the carbapenem genes of them by PCR methods.

RNA Isolation and Quantitative RT-PCR

The relative expression of $bla_{\text{KPC-2}}$ and capsule synthesis genes （*rmpA*, *rmpA2*, *magA*, *wzi*, and *manC*） were determined using quantitative RT-PCR method. Total RNA was isolated using Omega Bacteria RNA Isolation Kit (Omega). The qRT-PCR reactions were performed with performed with a Takara SYBR Premix Ex Taq Kit. The 2 −ΔΔCt (relative quantification) method was used to calculate the relative expression ratio. The primer sequences were presented in Supplementary.

Hypermucoviscosity sedimentation assays

The hypermucoviscosity was determined following a previously described methodology with minor modifications [5]. Briefly, bacteria were sub-cultured to an OD600 of 0.2 in LB broth at 37 °C with shaking. The samples were centrifuged at

10000g for 30s and the absorbance of supernatants was measured at OD600. Each assay was performed in duplicate and repeated three times independently.

Biofilm Formation Assay

Biofilm formation was performed as described previously in 96-well microtiter plates [6]. Briefly, 200μl of the mid-log phase bacteria cells ($1.5x10^7$ CFU/ml) was added to 96-well microtiter plates and incubated overnight. Next, all cultures were removed and then washed twice with phosphate-buffered saline. 0.1% crystal violet solution was used for staining for 20 min and washed three times. The biofilm formation was quantified by measuring the A_{590} after being solubilized with 200 μl of 95% ethanol. Each assay was performed in duplicate and repeated three times independently.

Serum resistance assay

The serum resistance was determined according to a previous study [6]. The mid-log phase bacteria cells were mixed with normal human serum at a 1:3 ratio and then incubated at 37°C for 2h. Serum resistance were characterized by plotting the average survival percentage of each strain against the incubation time. All assays were performed three times independently. *K. pneumoniae* strains were considered resistant to normal human serum if at least 90% of the organisms survived and were considered sensitive to serum if CFU counts dropped to 1% after 2 h of incubation.

Transmission electron microscopy

Transmission electron microscopy was performed using the Electron Microscopy Facility of Center for Excellence in Molecular Cell Science. A pellet of cells was fixed

overnight at 4°C in 2.5% glutaraldehyde. The pellet was washed twice in 0.1M phosphate buffer and postfixed with 1% osmium tetroxide for 1h, washed twice in 0.1M phosphate buffer water, and then washed twice in water and subsequent dehydration in grades of alcohol (15 min each: 30%, 50%, 70%, 80%, 95%; 2 × 10 min: 100%). The samples were then incubated twice with acetone for 15min and infiltrated overnight in a 1:1 mixture of acetone and Spurr's low-viscosity resin (Eponate 12). The following day the samples were embedded in Spurr's resin and polymerized at 60 °C for 48 h. The staining ultrathin sections (about 60 nm) were examined in a Tecnai G2 Spirit Twin transmission electron microscope and take corresponding images.

Whole genome sequencing and SNP analysis

Bacterial genomic DNA was extracted using TIANamp Bacteria DNA Kit according to manufacturer's recommendations. The bacteria whole genome resequencing was performed on Illumina NovaSeq platform. The GATK and SAMtools packages were used for SNP calling, and BWA software was used to map the raw paired-end reads onto the wild type reference genome [7]. The SNPs were annotated by ANNOVAR software and further determined by Sanger sequencing method [8].

Supplementary results

Diversity of TraM proteins

We identified 4391 TraM-carrying plasmids out of 14797 *Enterobacteriaceae* plasmids. The phylogenetic tree showed an abundant amino acid diversity of TraM proteins, and a total of 256 different TraM types were identified and were divided into several relatively clustered Clades (Figure S4B). Although $TraM_{pKPHS2}$ was located in

only a small branch, 605 of the 4391 *Enterobacteriaceae* plasmids carrying TraM had completely similar sequences to $TraM_{pKPHS2}$, and 588 of them were from *K. pneumoniae*, accounting for 11.5% (588/5095) of the total *K. pneumoniae* plasmids (Figure 3B). These results indicated that conjugative helper plasmids were widespread in *K. pneumoniae*, and these helper plasmids can also be detected in other *Enterobacteriaceae* bacteria.

References

1. Tian D, Wang M, Zhou Y, et al. Genetic diversity and evolution of the virulence plasmids encoding aerobactin and salmochelin in Klebsiella pneumoniae. Virulence. 2021 Dec;12(1):1323-1333.
2. Fu P, Tang Y, Li G, et al. Pandemic spread of blaKPC-2 among Klebsiella pneumoniae ST11 in China is associated with horizontal transfer mediated by IncFII-like plasmids. Int J Antimicrob Agents. 2019 Aug;54(2):117-124.
3. Tang Y, Fu P, Zhou Y, et al. Absence of the type I-E CRISPR-Cas system in Klebsiella pneumoniae clonal complex 258 is associated with dissemination of IncF epidemic resistance plasmids in this clonal complex. J Antimicrob Chemother. 2020 Apr 1;75(4):890-895.
4. Zhou Y, Tang Y, Fu P, et al. The type I-E CRISPR-Cas system influences the acquisition of blaKPC-IncF plasmid in Klebsiella pneumonia. Emerg Microbes Infect. 2020 Dec;9(1):1011-1022.
5. Ernst CM, Braxton JR, Rodriguez-Osorio CA, et al. Adaptive evolution of virulence and persistence in carbapenem-resistant Klebsiella pneumoniae. Nat Med. 2020 May;26(5):705-711.
6. Choi MJ, Ko KS. Loss of hypermucoviscosity and increased fitness cost in colistin-resistant Klebsiella pneumoniae sequence type 23 strains. Antimicrob Agents Chemother. 2015 Nov;59(11):6763-73.
7. McKenna A, Hanna M, Banks E, et al. The Genome Analysis Toolkit: a MapReduce framework for analyzing next-generation DNA sequencing data. Genome Res. 2010 Sep;20(9):1297-303.
8. Wang K, Li M, Hakonarson H. ANNOVAR: functional annotation of genetic variants from high-throughput sequencing data. Nucleic Acids Res. 2010 Sep;38(16):e164.

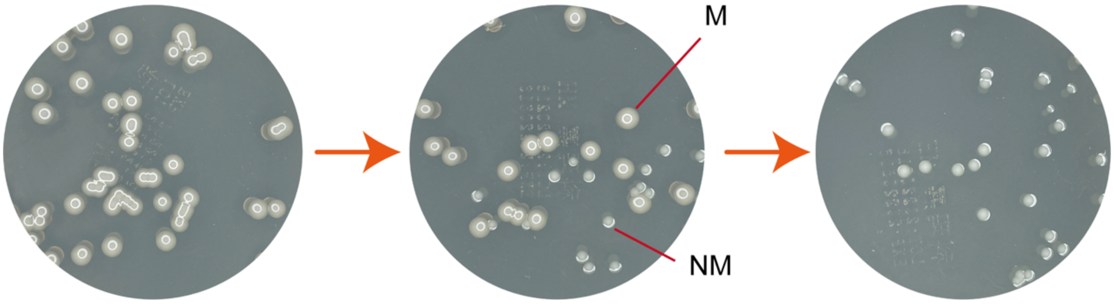

Figure S1. The mucoid and non-mucoid morphologies during passages

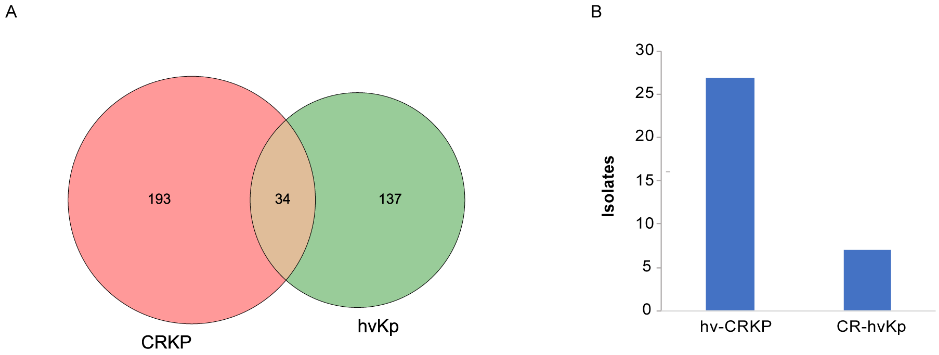

Figure S2. The distribution of clinical *Klebsiella pneumonaie.*

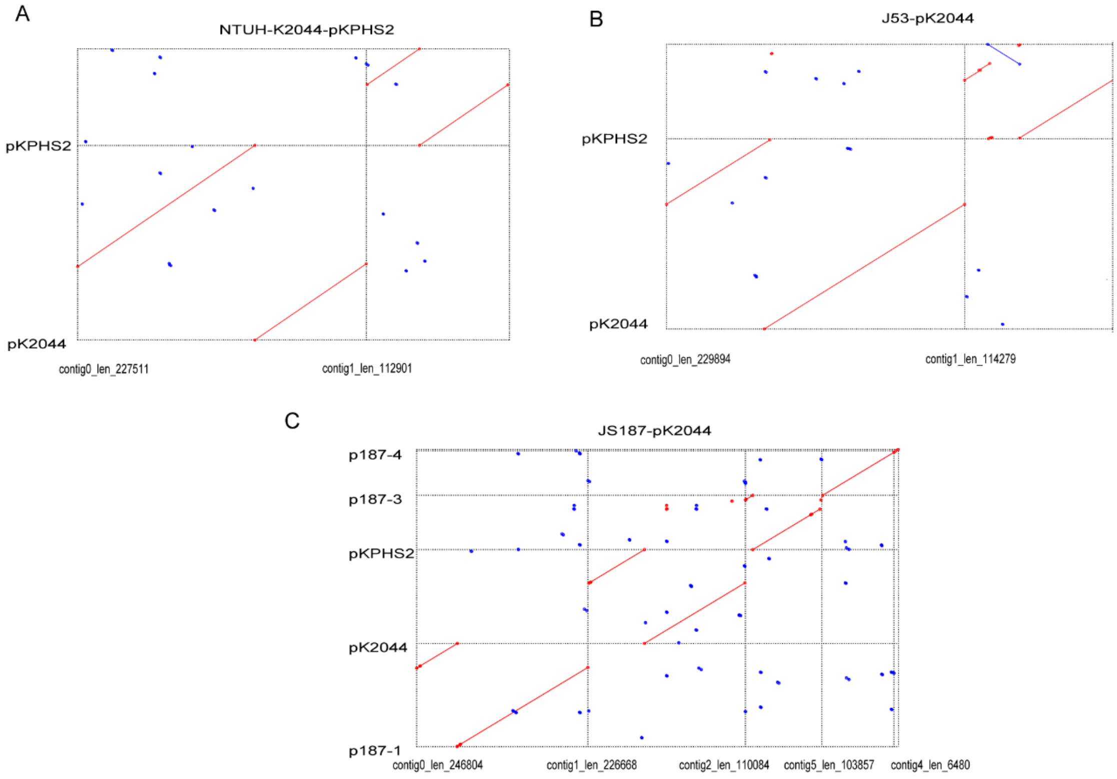

Figure S3. MUMmer-based comparison of plasmids of transconjugants and referenced plasmids. The abscissa indicates the plasmid contigs of transconjugants and the ordinate the reference plasmids.

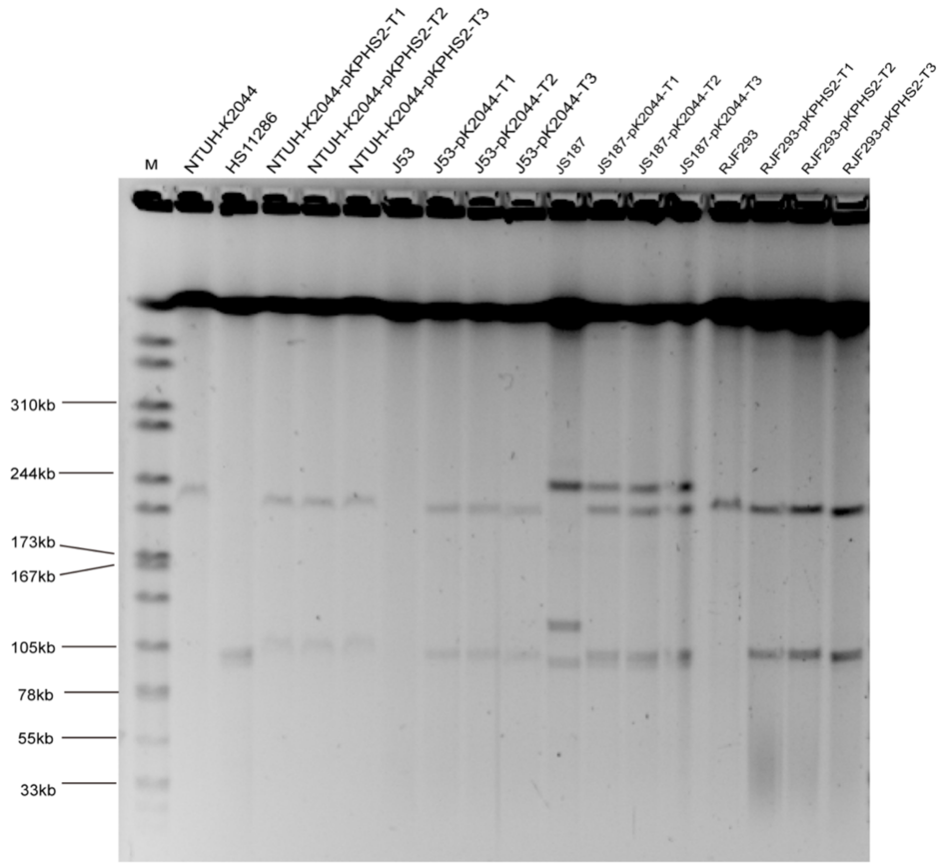

Figure S4 S1-PFGE profiles of transconjugants in this study.

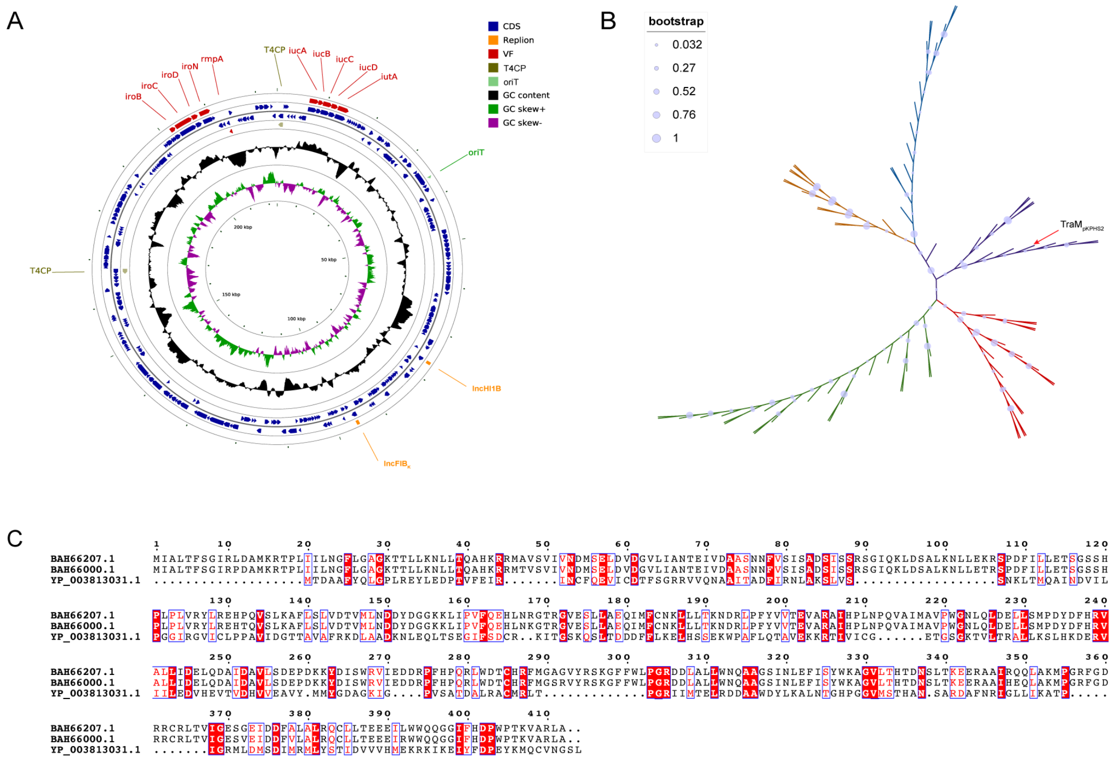

Figure S5. Characteristics of oriT and TraM. (A) The oriT and T4CPs were predicted by oriTfinder (https://bioinfo-mml.sjtu.edu.cn/oriTfinder/). (B) Diversity of TraM proteins. (C) Amino acid sequence alignment of two T4CPs in pK2044 (BAH66207.1, BAH66000.1) with reference T4CP (YP_003813031.1). Alignment was generated with the ClustalW multiple sequence alignment tool. Strictly conserved and highly conserved residues are highlighted in red boxes and red lettering, respectively.

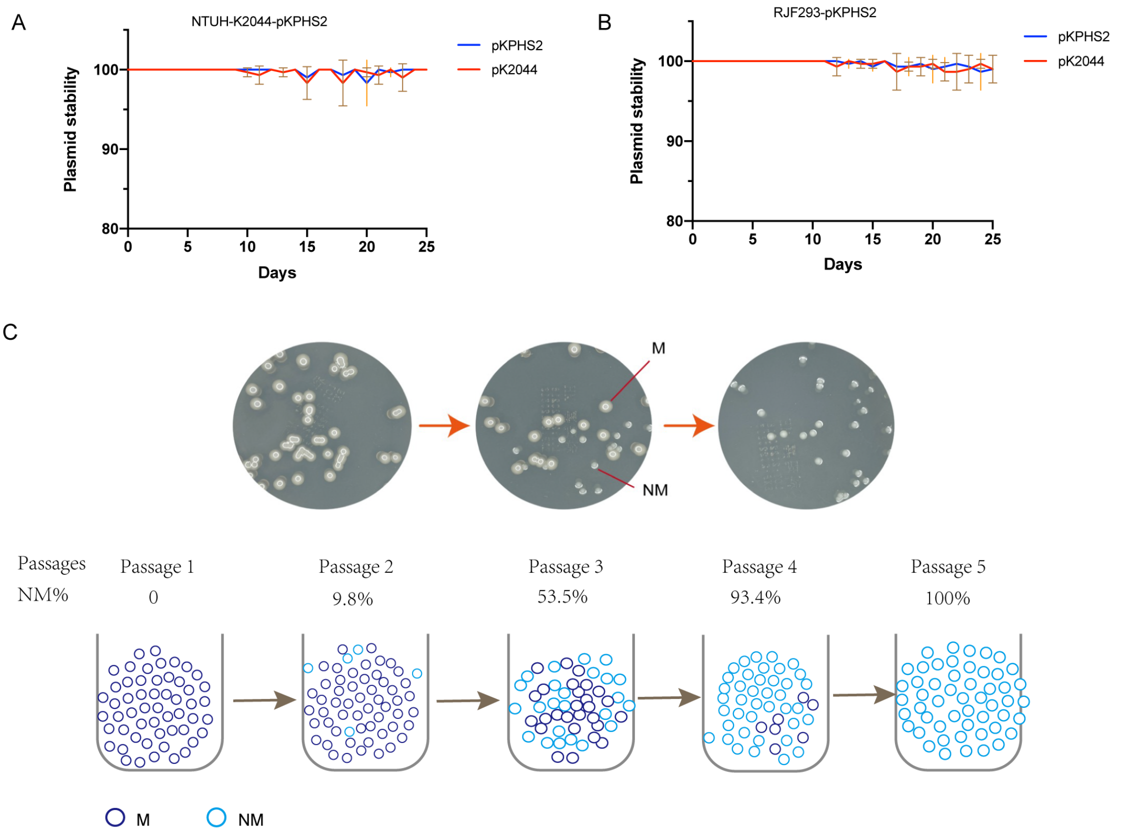

Figure S6. The stability of KPC plasmid and phenotypic changes of tranconjugants. The plasmid stability of NTUH-K2044-pKPHS2(A) and RJF293-pKPHS2 (B) in serial 25 passages. (C) The switch from mocoid to non-mocoid after several passages.

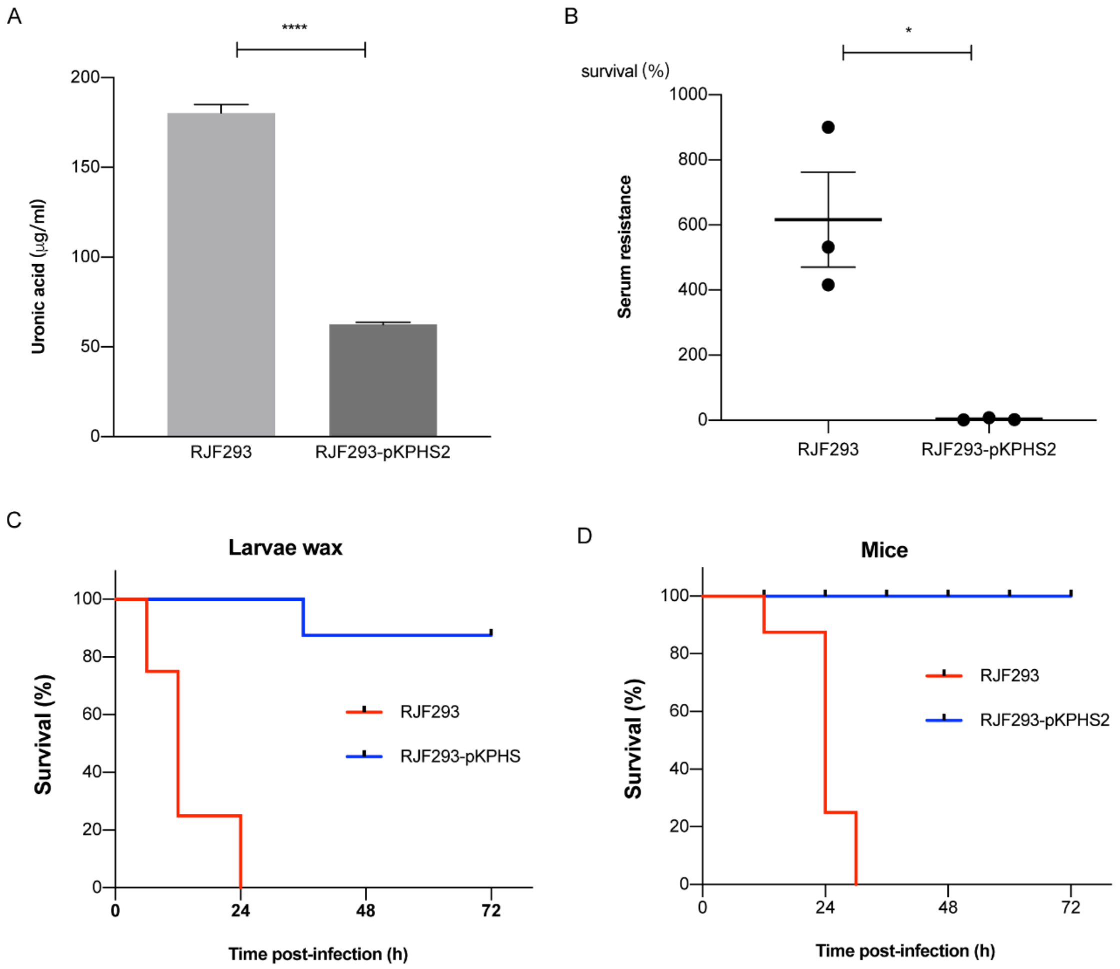

Figure S7. The virulence phenotypes and levels of K2-type CR-hvKp strain RJF293-pKPHS2. (A) Uronic acid. (B) Serum resistance. (C) The survival curves of infected Larvae wax. (D) The survival curves of infected mice. An unpaired two-sided Student's t-test was performed for uronic acid and serum resistance. A log-rank (Mantel–Cox) test was performed for the survival curves. ****$P < 0.0001$, *$P< 0.05$.

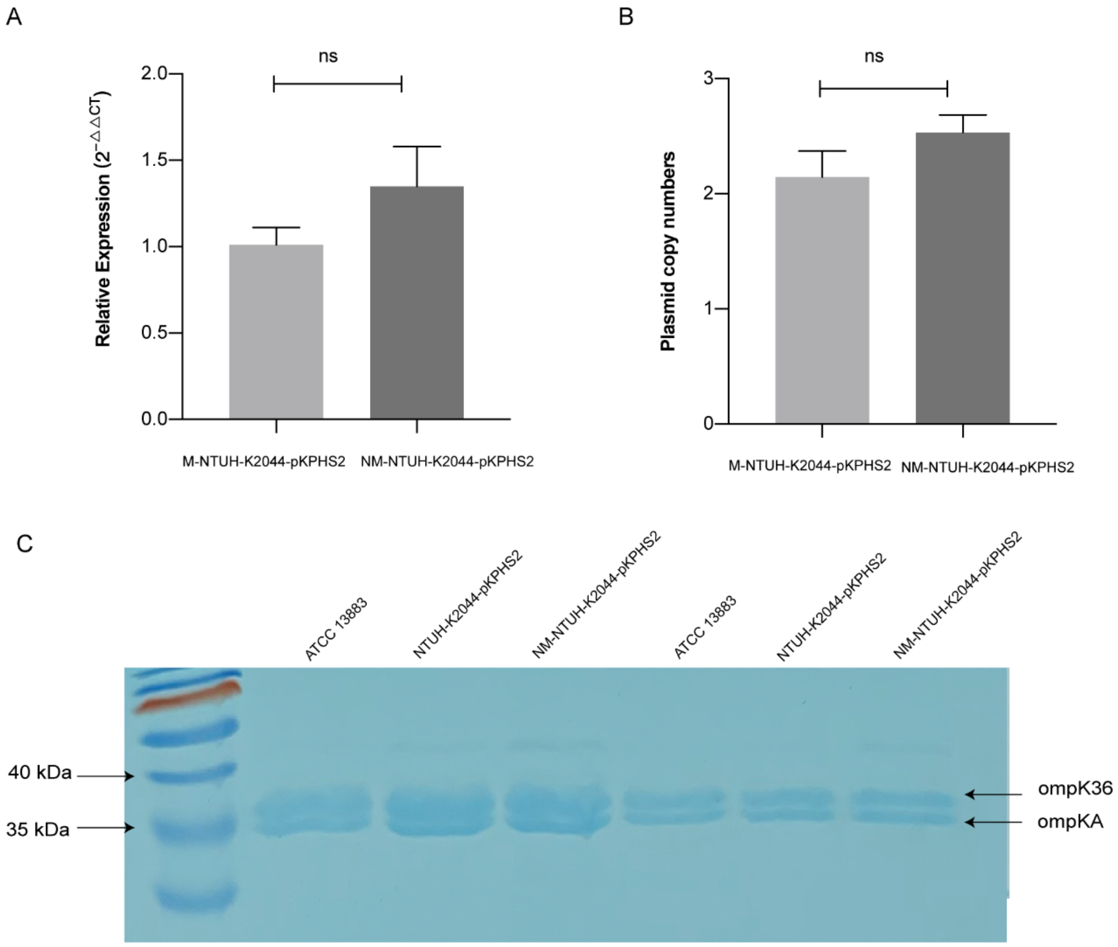

Figure S8. Some possible factors influencing carbapenem resistance. (A) The relative expression of *bla*$_{KPC-2.}$ (B) Plasmid copy numbers of KPC plasmid. A paired Student't test was performed. ns: not significant.
