# Supplementary material for: Prevalence of hypervirulent and carbapenem-resistant Klebsiella pneumoniae under divergent evolutionary patterns
Source: Emerg Microbes Infect. 2022 Aug 5;11(1):1936–49. doi: 10.1080/22221751.2022.2103454 (PMC9359173; doi:10.1080/22221751.2022.2103454)
Supplement: Supplemental Material [file TEMI_A_2103454_SM8825.zip › Supplementary_Materials/Supplementary_Table_S1.docx]

**Supplementary Table S1. Genomic characteristics of NTUH-K2044, HS11286, and JS187.**

| **Strain** | **NTUH-K2044** | **HS11286** | **JS187** |
| --- | --- | --- | --- |
| **MLST** | ST23 | ST11 | ST11 |
| **K-type** | KL1 | KL103 | KL103 |
| **Chromosome** | 5,248,520 bp | 5,333,942 bp | 5,359,967 bp |
| **Plasmids** | **pK2044**: IncFIB_K_/IncHI1B, 224152 bp | **pKPSH1**: IncFIB_pKPSH1,_ 122799 bp;  **pKPSH2**: IncFII_K_/IncR, 11195 bp;  **pKPSH3**: IncA/C, 105974 bp;  **pKPSH4**: Col156, 3751 bp;  **pKPSH5**: ColpHAD28, 3353 bp;  **pKPSH6**: ColpKPSH6, 1308 bp. | **p187-1**: IncFIB_pKPSH1,_ 246557 bp;  **p187-2**: IncFII_K_/IncR, 129684 bp;  **p187-3**: ColpHAD28, 3353 bp;  **p187-4**: IncA/C, 106402 bp. |
| **Resistance determinants** | **β-lactam**: SHV-11; **fosfomycin**: FosA6; **quinolone**: oqxA | **β-lactam**: KPC-2, SHV-11, CTX-M-14, TEM-1; **aminoglycoside**: rmtB, ANT(3'')  , AAC(3), APH(6), APH(3''); **sulphonamide**: sul2; **tetracycline**: tet(D); **fosfomycin**: FosA6. | **β-lactam**: KPC-2, SHV-11, SHV-64, CTX-M-14, CTX-M-3, TEM-1; **aminoglycoside**: ANT(3''), AAC(6'), APH(3'), APH(6), APH(3''), ANT(3''),AAC(3), rmtB; **quinolone**: oqxA, oqxB; **sulphonamide**: sul2; **tetracycline**: tet(D); **phenicol**: catB2; **fosfomycin**: FosA6, FosA3; **trimethoprim**: dfrA12. |
| **Virulence determinants** | *rmp*A, *rmp*A2, *iuc*ABCD-*iut*A, *iro*ABCD-*iro*N. | None | None |
