# Supplementary material for: Prevalence of hypervirulent and carbapenem-resistant Klebsiella pneumoniae under divergent evolutionary patterns
Source: Emerg Microbes Infect. 2022 Aug 5;11(1):1936–49. doi: 10.1080/22221751.2022.2103454 (PMC9359173; doi:10.1080/22221751.2022.2103454)
Supplement: Supplemental Material [file TEMI_A_2103454_SM8825.zip › Supplementary_Materials/Supplementary_Table_S6.docx]

**Supplementary Table S6 Primers used in this study**

| **Name** | **Sequences (5’-3’)** |
| --- | --- |
| **For screening transconjugants** | |
| iucA-F | GCTTATTTCTCCCCAACCC |
| iucA-R | TCAGCCCTTTAGCGACAAG |
| KPC-F | CTGTCTTGTCTCTCATGGCC |
| KPC-R | CCTCGCTGTGCTTGTCATCC |
| oqxA-F | CCAAAGTGACCGCCCCTATT |
| oqxA-R | GACGATGACGCTATCCCCAG |
| ICE-F | GGTGAGCTGACGAATGGATT |
| ICE-R | TTTCTCGGCACCAACTCAAA |
| **For the construction of pACYC-hyg-oriT_pK2044_ and pACYC-hyg** | |
| pACYC-F | ATGGAAGCCGGCGGC |
| pACYC-R | ACACGGTGCCTGACTGC |
| Hyg-F | GCAGTCAGGCACCGTGTATCCCCTGATTCCCTTTGTCAACAGC |
| Hyg-R (for pACYC-hyg-oriT_pK2044)_ | AGAGAGCCACTGCGGGATCGT |
| Hyg-R (for pACYC-hyg_)_ | GCCGCCGGCTTCCATAGAGAGCCACTGCGGGATCGT |
| oriT-F | ACGATCCCGCAGTGGCTCTCTTGGCTGTCCACATACTGGT |
| oriT-R | GCCGCCGGCTTCCATTGCGCTCAGCGATTTCACA |
| **For the knockout of oriT_pK2044_** | |
| Up-oriT-F | CCAGCCAGACGGTGTCATAA |
| Up-oriT-R | CTTTCTACGTGTTCCGCTTCCTTTAGCAGATAGCGGGCTGACGAAT |
| Kana-F | CTAAAGGAAGCGGAACACGTAGAAAG |
| Kana-R | ATTCCGAAGCCCAACCTTTCATAGA |
| Down-oriT-F | TCTATGAAAGGTTGGGCTTCGGAATTGGGGTTCGATCTATTGCCG |
| Down-oriT-R | ATACCGGACGCTGCTAGTTG |
| oriT-F（For screening） | TGCGCTCAGCGATTTCACA |
| oriT-R（For screening） | TGGCTGTCCACATACTGGT |
| **For screening carbapenem resistance genes** | |
| KPC-F | TCGCTAAACTCGAACAGG |
| KPC-R | TTACTGCCCGTTGACGCCCAATCC |
| NDM-F | GTTTGGCGATCTGGTTTTC |
| NDM-R | CGGAATGGCTCATCACGATC |
| OXA48-F | GCGTGGTTAAGGATGAACAC |
| OXA48-R | CATCAAGTTCAACCCAACCG |
| IMP-F | GGAATAGAGTGGCTTAAYTCTC |
| IMP-R | GGTTTAAYAAAACAACCACC |
| VIM-F | GATGGTGTTTGGTCGCATA |
| VIM-R | CGAATGCGCAGCACCAG |
| **For qPCR** | |
| qKPC-F | CAGTCGGAGACAAAACCGGA |
| qKPC-R | TCGCTGTGCTTGTCATCCTT |
| rpoB-F | ATATGATCAACGCCAAGCCG |
| rpoB-R | ATCTCAGACAGCGGGTTGTT |
| gyrB-F | CTGATTGCCGTGGTATCCGT |
| gyrB-R | CTCCACCGCTGATTTCACCT |
| **For construction of complement plasmid pACYC-rfaH** | |
| pACYC-rfaH-F | GCAGTCAGGCACCGTGTATGCAAGCCTGGTACT |
| pACYC-rfaH-R | GCCGCCGGCTTCCATTTAGATTTTCTGGAAGTCGGT |
| pACYC184-F | ATGGAAGCCGGCGGC |
| pACYC184-R | ACACGGTGCCTGACTGC |
| **For the knockout of *rfaH*** | |
| rfaH-up-F | AAATCGCGTCTTCACGCTGG |
| rfaH-up-R | GCTGTTGACAAAGGGAATCAGGGGATCGTTGTAGCTGCCCACGTTT |
| Hyg-F | ATCCCCTGATTCCCTTTGTCAACAGC |
| Hyg-R | AGAGAGCCACTGCGGGATCGT |
| rfaH-down-F | ACGATCCCGCAGTGGCTCTCTTAAGCAAGTCCTGCAGAGCG |
| rfaH-down-R | GACTTCGTGATGGCGCTGTT |
