# Supplementary material for: Prevalence of hypervirulent and carbapenem-resistant Klebsiella pneumoniae under divergent evolutionary patterns
Source: Emerg Microbes Infect. 2022 Aug 5;11(1):1936–49. doi: 10.1080/22221751.2022.2103454 (PMC9359173; doi:10.1080/22221751.2022.2103454)
Supplement: Supplemental Material [file TEMI_A_2103454_SM8825.zip › Supplementary_Materials/Supplementary_Table_S7.docx]

**Supplementary Table S7 Strains and plasmids in this study**

| **Strains/Plasmids** | **Characteristics** | **Source** |
| --- | --- | --- |
| J53 | *E. coli,* sodium azide-resistant, recipient for plasmid conjugation | Xiaofei Jiang Laboratory |
| JS187 | ST11 *K. pneumoniae,* carried a *bla*_KPC-2_-positive plasmid (p187-2), Assembly: GCA_002848565.1 | Xiaofei Jiang Laboratory |
| HS11286 | ST11 *K. pneumoniae,* carried a *bla*_KPC-2_-positive plasmid (pKPHS2), Assembly: GCA_000240185.2 | Xiaofei Jiang Laboratory |
| NTUH-K2044 | ST23 *K. pneumoniae,* K1 type hypervirulent strain, isolated from a patient with liver abscess, carried a virulence plasmid pK2044, Assembly: GCA_000009885.1 | Xiaofei Jiang Laboratory |
| RJF293 | ST374 *K. pneumoniae,* K2 type hypervirulent strain, carried a virulence plasmid，Assembly: GCA_001530015.1 | Xiaofei Jiang Laboratory |
| NTUH-K2044-pKPHS2 | NTUH-K2044 obtained the *bla*_KPC-2_-positive plasmid pKPHS2 | This study |
| RJF293-pKPHS2 | RJF293 obtained the *bla*_KPC-2_-positive plasmid pKPHS2 | This study |
| JS187-vir-pK2044 | CRKP strain JS187 obtained virulence plasmid pK2044 and KPC plasmid pKPHS2 | This study |
| J53-vir-pK2044 | *E. coli* J53 obtained virulence plasmid pK2044 and KPC plasmid pKPHS2 | This study |
| HS11286-pACYC-Hyg | HS11286 carried empty vector pACYC-Hyg | This study |
| HS11286-pACYC-Hyg-oriTpK2044 | HS11286 carried mimic virulence plasmid pACYC-Hyg-oriTpK2044 | This study |
| J53-pACYC-Hyg-oriT_pK2044_ | *E. coli* J53 carried mimic virulence plasmid pACYC-Hyg-oriTpK2044 | This study |
| NTUH-K2044∆magA | NTUH-K2044 was knocked out of the *magA* gene | This study |
| NTUH-K2044∆magA-pKPHS2 | NTUH-K2044∆magA obtained the KPC plasmid pKPSH2 | This study |
| NTUH-K2044∆magA∆rfaH-pKPHS2 | NTUH-K2044∆magA-pKPHS2 was knocked out of the *rfaH* gene | This study |
| NTUH-K2044∆magA∆rfaH-pKPHS2-pACYC-rfaH | NTUH-K2044∆magA∆rfaH-pKPHS2 was complemented with plasmid pACYC-rfaH | This study |
| NM-NTUH-K2044-pKPHS2-pACYC-rfaH | NM-NTUH-K2044-pKPHS2 was complemented with plasmid pACYC-rfaH | This study |
| RJF293∆rfaH-pKPHS2 | RJF293-pKPHS2 was knocked out of the *rfaH* gene | This study |
| RJF293∆rfaH-pKPHS-pACYC-rfaH | RJF293∆rfaH-pKPHS2 was complemented with plasmid pACYC-rfaH | This study |
| NTUH-K2044-∆oriT_pK2044_ | NTUH-K2044 was knocked out of the oriT_pK2044_ | This study |
| **Plasmid** | | |
| pACYC184 | CAMR TerR, ori p15A, low copy, vector for plasmid construction | Xiaofei Jiang Laboratory |
| pACYC-Hyg | CAMR Hyg^R^, ori p15A, low copy, vector for plasmid construction | This study |
| pACYC-Hyg-oriT_pK2044_ | CAMR Hyg^R^，ori p15A, low copy, oriT_pK2044_ sequence was introduced into pACYC-Hyg | This study |
| pACYC-rfaH | CAMR Hyg^R^，ori p15A, low copy, *rfaH* gene was introduced into vector pACYC184 to complement of *rfaH* | This study |
| pKOBEG | Apr^R^, plasmid encode recombinant proteins for λ-*Red* homologous recombination，30°C | Xiaofei Jiang Laboratory |
